# NAC and Zuotin/Hsp70 chaperone systems coexist at the ribosome tunnel exit *in vivo*

**DOI:** 10.1093/nar/gkae005

**Published:** 2024-01-15

**Authors:** Thomas Ziegelhoffer, Amit K Verma, Wojciech Delewski, Brenda A Schilke, Paige M Hill, Marcin Pitek, Jaroslaw Marszalek, Elizabeth A Craig

**Affiliations:** Department of Biochemistry, University of Wisconsin—Madison, Madison, WI 53726, USA; Department of Biochemistry, University of Wisconsin—Madison, Madison, WI 53726, USA; Department of Biochemistry, University of Wisconsin—Madison, Madison, WI 53726, USA; Department of Biochemistry, University of Wisconsin—Madison, Madison, WI 53726, USA; Department of Biochemistry, University of Wisconsin—Madison, Madison, WI 53726, USA; Intercollegiate Faculty of Biotechnology, University of Gdansk and Medical University of Gdansk, Gdansk 80-307, Poland; Department of Biochemistry, University of Wisconsin—Madison, Madison, WI 53726, USA; Intercollegiate Faculty of Biotechnology, University of Gdansk and Medical University of Gdansk, Gdansk 80-307, Poland; Department of Biochemistry, University of Wisconsin—Madison, Madison, WI 53726, USA

## Abstract

The area surrounding the tunnel exit of the 60S ribosomal subunit is a hub for proteins involved in maturation and folding of emerging nascent polypeptide chains. How different factors vie for positioning at the tunnel exit in the complex cellular environment is not well understood. We used *in vivo* site-specific cross-linking to approach this question, focusing on two abundant factors—the nascent chain-associated complex (NAC) and the Hsp70 chaperone system that includes the J-domain protein co-chaperone Zuotin. We found that NAC and Zuotin can cross-link to each other at the ribosome, even when translation initiation is inhibited. Positions yielding NAC–Zuotin cross-links indicate that when both are present the central globular domain of NAC is modestly shifted from the mutually exclusive position observed in cryogenic electron microscopy analysis. Cross-linking results also suggest that, even in NAC’s presence, Hsp70 can situate in a manner conducive for productive nascent chain interaction—with the peptide binding site at the tunnel exit and the J-domain of Zuotin appropriately positioned to drive stabilization of nascent chain binding. Overall, our results are consistent with the idea that, *in vivo*, the NAC and Hsp70 systems can productively position on the ribosome simultaneously.

## Graphical Abstract

**Graphical Abstract:**
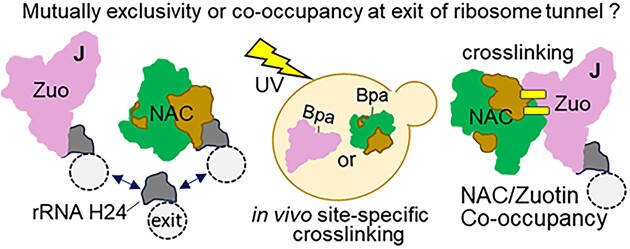

## Introduction

Thousands of different nascent chains exit the ribosome tunnel. They differ widely in amino acid sequence, each with their own inherent challenges for folding or transport to organelles (1). The area surrounding the tunnel exit is a hub for interaction of factors important for protein maturation and transport (2), raising the question of how these different proteins are accommodated in the crowded cellular environment. Two particularly abundant molecular chaperone systems of eukaryotes bind both translating and nontranslating ribosomes and interact with a wide variety of nascent chains—a ribosome-based Hsp70 system and the nascent chain-associated complex (NAC) (3,4).

NAC is a stable heterodimer, formed by interaction of two related subunits: ∼19 kDa NACα and ∼17 kDa NACβ (5). Internal segments form the core globular domain—antiparallel N-terminal helices, followed by five β-strands each (6,7). The globular domains of both NACα and NACβ have N- and C-terminal extensions. A positively charged cluster of residues in NACβ’s N-terminal extension is critical for ribosome association via interaction with ribosomal RNA (rRNA) (8,9). In recent cryogenic electron microscopy (cryo-EM) studies, this N-terminal segment was found to be situated near ribosomal proteins eL22 and eL19, with the globular domain positioned nearer the tunnel exit, abutting the tip of rRNA helix 24 (H24) (10).

Like all Hsp70s (11–13), the ribosome-based system is ATP-dependent, requiring hydrolysis of Hsp70-bound ATP to drive the massive conformational changes required to stabilize interaction with substrate proteins (4). Hydrolysis is driven by synergistic action of a J-domain protein co-chaperone and a substrate polypeptide transiently interacting in the substrate binding cleft (14). The J-domain protein of the ribosome system, which is generically referred to as Zuotin, is called Zuo1 in *Saccharomyces cerevisiae* (15). It partners with the Hsp70 Ssb, which is encoded by two highly conserved paralogous genes, *SSB1* and *SSB2*. Zuo1 binds the ribosome close to the tunnel exit via its Zuotin homology domain (ZHD) (4,16,17). The tip of rRNA H24 is critical for interaction (16). The N-terminus of Zuo1 binds the atypical Hsp70 Ssz1 (18), forming an extremely stable heterodimer (often called the ribosome-associated complex, RAC). Ssz1 binds transiently to Ssb(ATP) (19), recruiting it to the ribosome.

Both RAC and NAC are abundant and bind both translating and nontranslating ribosomes (20). The ratios of NAC and RAC to ribosomes are ∼1:1 and between 0.3 and 0.5:1, respectively (20,21). Although some data suggest that NAC and RAC can coexist at the ribosome (22), more recent structural and biochemical evidence indicates that their binding to the ribosome is mutually exclusive (10,23). These results raise the question as to how these ribosome-associated systems are able to effectively join forces to facilitate nascent chain folding *in vivo*. To begin to address this question, we utilized *in vivo* site-specific cross-linking to assess whether, in the cell, the Hsp70 system and NAC can occupy a ribosome at the same time, and if they do, their relative positionings.

## Materials and methods

### Strains and plasmids

*Saccharomyces cerevisiae* strains and plasmids used are listed in Supplementary Tables S1 and S2, respectively. All DNA primers and gBlocks were synthesized by Integrated DNA Technologies (Coralville, IA). Sequencing reactions were analyzed by Functional Biosciences (Madison, WI). Yeast cells were grown in YPD [1% yeast extract, 2% peptone (Difco Laboratories, Detroit MI), 2% dextrose] or selective minimal media [0.67% yeast nitrogen base without amino acids (US Biological, Marblehead, MA), 2% dextrose], supplemented with required amino acids (24). Transformations were carried out using a previously developed protocol (25).

*EGD1* and *EGD2*, which encode NACβ and NACα, respectively, were deleted using CRISPR. Target-specific single-guide RNAs (sgRNAs) were created by using 60-mer bridging primers, synthesized by Integrated DNA Technologies (Coralville, IA), containing a 20-nucleotide target sequence of +69 to +88 for *EGD1* and +465 to +484 for *EGD2*, which were cloned into NotI-digested pXIPHOS vector (accession MG897154, GenBank) (26,27) using NEBuilder HiFi DNA Assembly Master Mix (New England Biolabs). Rescue DNAs were synthesized as gBlocks consisting of 200 bp upstream of the ATG start codon followed by 200 bp downstream of the stop codon for both *EGD1* and *EGD2*. The pXIPHOS-*EGD1* (or *EGD2*) sgRNA plasmid, which carries the natamycin resistance marker, was co-transformed into yeast with a 20× molar excess of rescue DNA. Natamycin-resistant transformants were selected on YPD with 100 μg/ml nourseothricin (Werner BioAgents GmbH, Jena, Germany). Transformants were tested by colony polymerase chain reaction (PCR) using primers that bind within the upstream and downstream untranslated regions (UTRs). The TAG stop codon of *SSZ1* was replaced with TAA using CRISPR. A target-specific sgRNA was created by cloning a 60-mer bridging primer containing *SSZ1* sequence from +1461 to +1442 into pXIPHOS. Rescue DNA was synthesized as a gBlock from +1311 to +1748 with the TAG to TAA change and a codon change for Ser480 to mutate Cas9 cleavage site. Yeast were co-transformed with pXIPHOS-*SSZ1* and rescue DNA. Genomic DNA was isolated from natamycin-resistant transformants and the *SSZ1* gene was PCR amplified followed by sequencing to confirm the TAG to TAA change.

To allow depletion of translation initiation factor eIF3b encoded by *PRT1*, a truncated degron tag, AID^71–114^, was added to *PRT1* immediately prior to its stop codon using CRISPR (28). The target-specific sgRNA contains *PRT1* sequence from +2241 to +2222. The rescue DNA consists of 125 bp of *PRT1* sequence upstream of the stop codon followed by 132 bp encoding AID^71–114^ and ending with 171 bp of downstream *PRT1* UTR sequence. Yeast strains containing PRT1–AID^71–114^ were verified using similar methods as described above for *SSZ1*-TAG/TAA.

For *in vivo* cross-linking, Egd1 (NACβ) and Egd2 (NACα) open reading frames were placed under the control of the glycerol-3-phosphate dehydrogenase (GPD) promoter (29) to generate plasmids p415GPD-*EGD1* and p416GPD-*EGD2*.

### *In vivo* cross-linking

All yeast strains to be used for *in vivo* cross-linking were transformed with plasmid ptRNA-Bpa, which directs incorporation of *p*-benzoyl-l-phenylalanine (Bpa) at TAG codons, and a plasmid having a TAG mutation at the desired position for Bpa incorporation into the target protein. Strains were then grown on the appropriate selective minimal medium with 0.4 mM Bpa, starting at an OD_600nm_ between 0.03 and 0.10. At OD_600nm_ = 0.5–1.0, cycloheximide was added to 0.1 mg/ml and cells harvested by centrifugation. 12.5–25 OD_600nm_ units of resuspended cells were divided in half—one half was subjected to 365 nm UV illumination for 1 h at 4°C (Stratalinker 1800 UV cross-linker with 365 nm bulbs) and the other was kept on ice as a control. Cells were lysed by agitation with glass beads for 5 min at 4°C in lysis buffer [300 mM sorbitol, 20 mM HEPES–KOH, pH 7.5, 1 mM EGTA, 5 mM MgCl_2_, 10 mM KCl, 10% (v/v) glycerol, 1 mM dithiothreitol and RNasin RNase Inhibitor (Promega) at a dilution of 1:1000]. Lysates were clarified by centrifugation at 16 100 rcf for 10 min in a microcentrifuge (Eppendorf). Typically, 5 Abs_260_ units of cleared cell lysate was loaded onto a 0.8 ml sucrose cushion composed of lysis buffer having 0.5 M sucrose instead of sorbitol. To pellet ribosomes, the cell extracts were centrifuged for 2 h at 150 700 rcf in a MLA130 rotor (Beckman Coulter) at 4°C. The pellet was suspended in sodium dodecyl sulfate (SDS) sample buffer [0.124 M Tris–HCl buffer, pH 6.8, 4% (w/v) SDS, 10% (v/v) glycerol, 0.02% bromophenol blue, 4.5% β-mercaptoethanol] and used for immunoblotting. Three or more independent strains were analyzed with similar results, for each positive Bpa variant reported. We note that the level of expression of Bpa-containing constructs can vary depending on the position of the TAG codon.

For glucose starvation experiments, the experimental culture was pelleted and resuspended in prewarmed minimal medium lacking glucose and supplemented with 2% glycerol and 2% ethanol. Incubation was continued for 10 min before processing. For depletion of eIF3b encoded by *PRT1*, duplicate cultures of PRT1–AID^71–114^ strains were prepared; one was treated with 200 μM 1-naphthaleneacetic acid (NAA) for 60 min prior to processing. Effectiveness of the degron at eliminating eIF3b activity was tested by monitoring growth on complete minimal media plates containing 250 μM of NAA (Sigma–Aldrich, St Louis, MO). It was empirically determined that maximum collapse of polysomes occurred within 60 min in 200 μM NAA for cells grown in liquid defined minimal media. For polysome analysis, 8 Abs_260_ units of extract was applied to 10–50% sucrose gradients (17). After centrifugation, gradients were fractionated and monitored for absorbance at 254 nm to detect nucleic acids.

### Immunoblot analysis

Resuspended pellets from sucrose cushions were resolved on SDS–PAGE gels (7.5% acrylamide, unless otherwise indicated) and subjected to immunoblot analysis. Two prestained size markers BlueEye (Sigma) and PageRuler (Fisher) differing in mobility in the 70–120 kDa range were used. In Figure 5C, both are shown on the same blot for comparison—BlueEye on left and PageRuler on right. Rabbit polyclonal antibodies were used throughout. Antibodies specific for uL29 were kindly provided by Sabine Rospert (20,30). Antibodies specific for Ssz1 (31), Ssb (32) and Zuo1 (16) were previously described. Antibodies specific for NACα or NACβ were obtained from rabbits using purified proteins as antigens (Harlan Laboratories, Inc.). NACα or NACβ were expressed and purified as N-terminal His_6_-*Smt3* (SUMO) fusion proteins (19,33) using HisPur Ni-NTA Resin (Thermo Scientific) as recommended by the manufacturer, followed by removal of the fusion tag by treatment with Ulp protease. As NACβ was relatively insoluble, initial binding to Ni-NTA resin was performed under denaturing conditions (6 M guanidine hydrochloride) and washed with 8 M urea in 20 mM NaHPO_3_ (pH 7.8) and 0.5 M NaCl. The resin was then washed with NAC buffer [25 mM HEPES, pH 7.5, 150 mM NaCl, 10 mM KCl, 5 mM MgCl_2_, 25 mM imidazole, 5 mM 2-mercaptoethanol, 5% (v/v) glycerol] prior to elution with NAC buffer plus 0.3 M imidazole.

### Mass spectrometry

Resuspended sucrose cushion pellets were resolved on 7.5% SDS–PAGE, followed by staining with either Coomassie brilliant blue or Pierce™ (Thermo Scientific) silver stain for mass spectrometry. After band excision and destaining, samples were alkylated with iodoacetic acid prior to digestion with trypsin and solid phase extracted as described previously (21). Resulting peptides were analyzed by nanoscale liquid chromatography coupled to tandem mass spectrometry using the Agilent 1100 nanoflow system (Agilent) connected to a hybrid linear ion trap-orbitrap mass spectrometer (LTQ-Orbitrap Elite™, Thermo Fisher Scientific) equipped with an EASY-Spray™ electrospray source (21). Raw tandem mass spectrometry data were converted to mgf file format using MSConvert (ProteoWizard: open source software for rapid proteomics tools development). To identify the proteins most highly represented in each sample, Scaffold4 (Proteome Software Inc.) was used to rank candidates based on total spectrum count.

### Molecular modeling

*Saccharomyces cerevisiae* Zuo1 structural model (AlphaFold AF-P32527-F1) (34,35) and ribosome structure (PDBID: 6TNU) (36) were fitted into the cryo-EM map of a ribosome-nascent chain (RNC) complex with Zuo1 and Ssz1 bound (EMDB: EMD-32977) (37) using ChimeraX ‘fit in map’ function (38), yielding the Zuo1–ribosome structural model (ribosome–Zuo1) used throughout. To position the *S. cerevisiae* NACα–NACβ globular domain (NACα N14-T75 and NACβ D40-L118), structural model (ModelArchive, DOI: 10.5452/ma-bak-cepc) (39) was first oriented manually in PyMOL to accommodate both the NAC–Zuo1 cross-links and the known association of the N-terminus of NACβ with the ribosome. Next, NAC alone and Zuo1 with nearby side chains of ribosomal components were independently relaxed by performing 100 runs of FastRelax protocol in PyRosetta (40) using REF2015 energy function. Five top-scoring poses of NAC and Zuo1 with nearby ribosomal components were then combinatorically merged. Twenty-five resulting models were locally docked 200 times, each in high-resolution mode in PyRosetta by applying DockMCMProtocol mover (41). The top 5% of best-scoring poses were filtered by (i) compatibility of the N-terminus of NACβ globular domain with NACβ N-terminal ribosome anchoring extension derived from cryo-EM structure of RNC–NAC complex (PDBID: 7QWR) (10) and (ii) satisfaction of cross-links, as described in (42). The final model was obtained from the pose satisfying the greatest fraction of cross-links by rebuilding the anchoring linker (residues A31–K39) between the extreme N-terminal NACβ helix and the NAC globular domain using PyRosetta LoopModeler. The Ssb1–Zuo1 complex was predicted using ColabFold (v1.5.2) implementation (43) of AlphaFold-Multimer-v3 (44) and positioned on the ribosome by fitting the Zuo1/ribosome structure (PDBID: 6TNU) to the cryo-EM map of RNC complex in complex with Zuo1 and Ssz1 (EMDB: EMD-32977). All structural visualizations were prepared by Visual Molecular Dynamics (39) and Blender (https://www.blender.org/).

## Results and discussion

### Hsp70 Ssb1 cross-links to NAC *in vivo*

**Figure 1.**
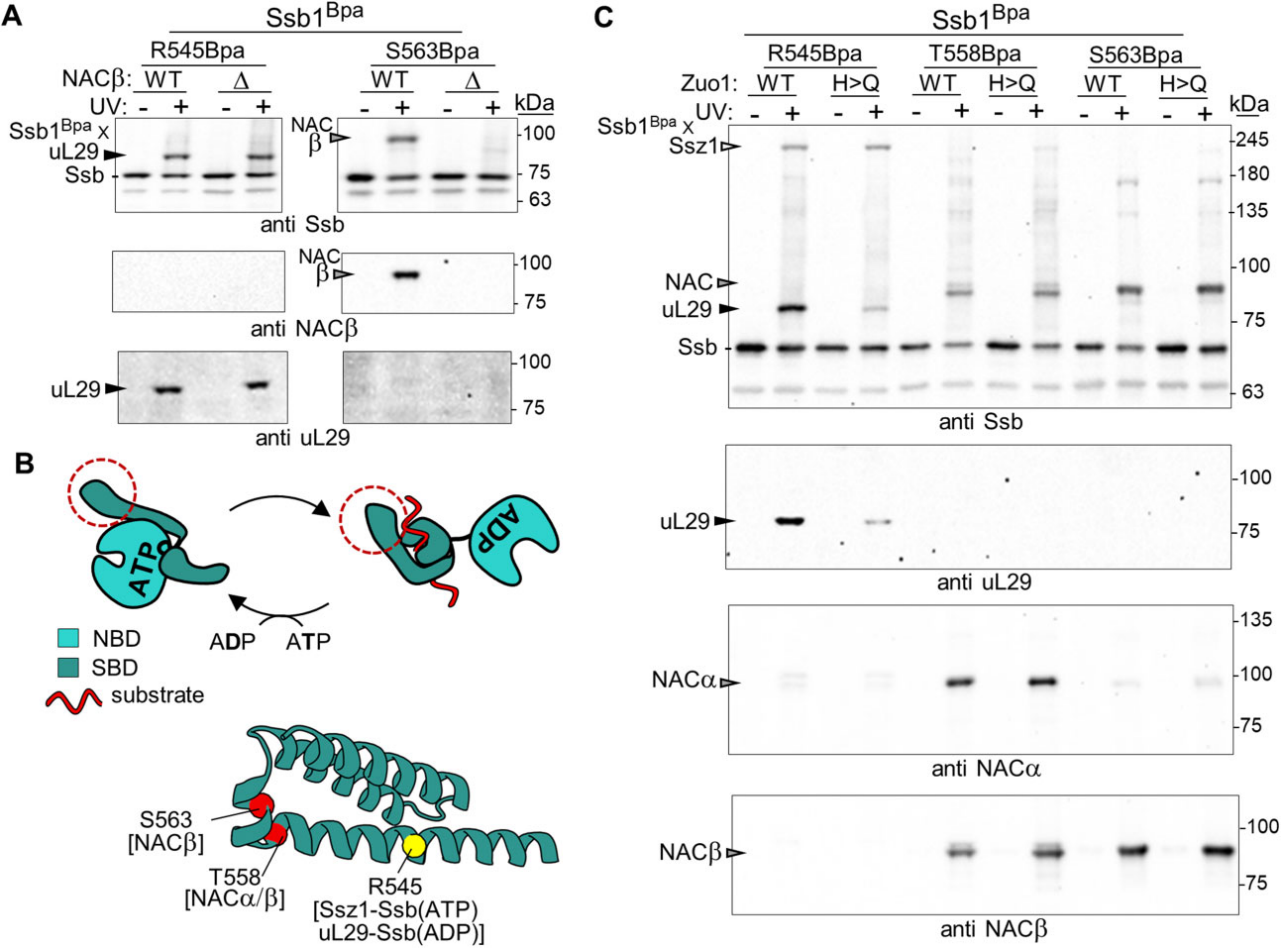
Hsp70 Ssb1 cross-links to NAC *in vivo*. (A and C) Cells expressing Ssb1 variants with Bpa incorporated at indicated positions were exposed to UV light (+) or left unexposed (−). Cross-linking was analyzed by immunoblotting after SDS–PAGE using antibodies specific for Ssb, NACα, NACβ or uL29, as indicated (anti). Ssb1^Bpa^ cross-link products to NAC, Ssz1 and uL29 are indicated with arrowheads, and non-cross-linked proteins and migration of molecular weight markers (kDa) by dashes. WT, wild-type gene. (**A**) The two Ssb1 Bpa variants subjected to mass spectrometric analysis (see Supplementary Table S3) were compared using immunoblot analysis. Δ, deletion of *EGD1* gene encoding NACβ. 7.5-12% gradient gel was used. (**B**) Schematic of the ATP-dependent Hsp70 peptide binding cycle. Nucleotide binding domain (NBD); substrate binding domain (SBD). ATP hydrolysis results in dissociation of both SBD subdomains from the NBD, allowing the αSBD lid subdomain to close over the transiently bound substrate. Dotted circle indicates segment of αSBD shown at bottom—AlphaFold model (AF-P11484-F1) of Ssb1(ATP). Positions having Bpa incorporated in A and B shown as spheres with the cross-link partner in parentheses. (**C**) Immunoblot analysis of Bpa variants in WT *ZUO1* and *zuo1^H128Q^* (H>Q) backgrounds.

In earlier studies aimed at understanding the positioning of RAC and Hsp70 Ssb on ribosomes, we carried out *in vivo* site-specific cross-linking. Nonsense suppression was used to incorporate the noncanonical, photoactivatable amino acid Bpa in place of specific individual endogenous amino acids (16,19). Several relatively fast migrating Ssb1^Bpa^ cross-link products (i.e. at an apparent molecular weight of 85–95 kDa) were obtained when Bpa was inserted into Ssb1’s substrate binding domain (SBD) (19). Using immunoblot analysis, a number of them were determined to be ribosomal proteins (e.g. R545Bpa to 14 kDa uL29) (Figure 1A). However, one, which did not react with ribosomal protein antibodies, formed when Bpa was inserted at position S563—in the ‘lid’ subdomain of the SBD (called αSBD). Upon hydrolysis of ATP-bound Hsp70, the lid closes over the peptide binding site in the β subdomain of the SBD (βSBD) (Figure 1B). To identify this cross-link product, we performed mass spectrometry, including Ssb1^R545Bpa^ as a control. As expected, uL29 was the predominant small protein detected comigrating with the Ssb1^R545Bpa^ cross-link product. NACβ was the predominant comigrating small protein in the Ssb1^S563Bpa^ sample (Supplementary Table S3). That Ssb1^S563Bpa^ cross-links to NACβ was then verified by immunoblot analysis: the Ssb1^S563Bpa^ cross-link product migrating at ∼90 kDa, which was undetectable in a strain having a deletion of the gene encoding the predominant NACβ isoform, reacted with NACβ-specific antibodies (Figure 1A and Supplementary Figure S1). Subsequently, Bpa was incorporated at four nearby positions in Ssb1. Ssb1^T558Bpa^ cross-linked to both subunits of NAC (i.e. NACα and NACβ) (Figure 1C).

Ssb is present at the ribosome in both ATP- and ADP-bound states. Ssb(ATP) interacts with the Ssz1 subunit of RAC; after hydrolysis, Ssb(ADP) interacts directly with the ribosome, as indicated by its cross-linking to ribosomal proteins on the other side of the tunnel exit (e.g. uL24 and uL29) (19,45,46). Therefore, we next assessed which nucleotide state of Ssb1^Bpa^ was cross-linking to NAC. To do so, we compared cross-linking of Ssb1^T558Bpa^ and Ssb1^S563Bpa^ to NAC in cells expressing either WT Zuo1 or a variant defective in stimulation of Ssb’s ATPase activity, thus preventing efficient conversion of Ssb to the ADP-bound state. We used the well-characterized Zuo1^H128Q^ variant that has a substitution in the J-domain’s HPD motif crucial for ATPase stimulation (47). Ssb1^R545Bpa^ was included as a control, as it cross-links to both Ssz1 and uL29—Ssz1 cross-linking being similar in WT and *zuo1^H128Q^* cells, and uL29 cross-linking being less in *zuo1^H128Q^* cells than in WT cells (Figure 1C) (19). Cross-linking of Ssb1^S563Bpa^ and Ssb1^T558Bpa^ to NAC was similar in WT and *zuo1^H128Q^* cells (Figure 1C). We conclude that Ssb(ATP) and NAC are present on ribosomes concurrently. As Zuo1 is required for Ssb function, we investigated, as described below, whether Zuo1 and NAC are also on the ribosome at the same time.

### Zuo1 cross-links to NAC *in vivo*

**Figure 2.**
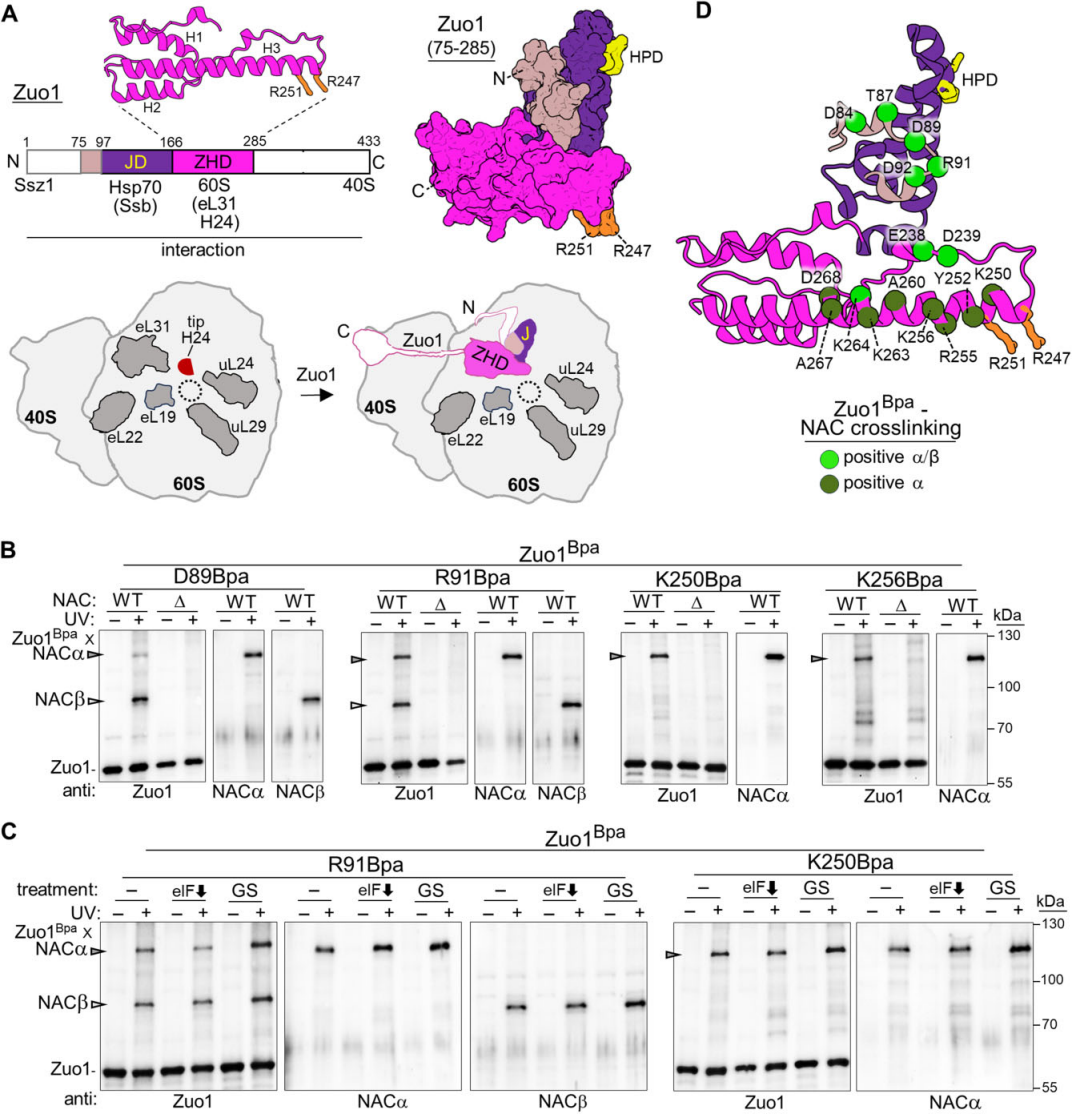
Zuo1^Bpa^ cross-links to NAC *in vivo*. (**A**) Zuo1 and its association with the ribosome. (Top left) Line diagram of Zuo1 domains, highlighting the more N-terminal regions that are positioned at the 60S subunit near the tunnel exit. J-domain (JD); Zuotin Homology Domain (ZHD), which associates directly with the 60S. Residue position above and interaction partner below diagram. ZHD structure (residues 166–285) shown as cartoon with the three helices indicated; two arginines required for H24 binding shown in stick representation. (Top right) Residues 75–285 of AlphaFold modeled structure of Zuo1 (AF-P32527-F1) in surface representation; colors as in the left panel, with critical HPD motif of JD needed for stimulation of Hsp70 ATPase activity and arginine residues (R247 and R251) known to be critical for ribosome association via interaction with H24 in stick representation. (Bottom) Schematic of the ribosome without (left) or with (right) Zuo1 bound. Select ribosomal proteins near tunnel exit (dotted circle) shown in dark gray; position of the tip of rRNA H24 indicated. Zuo1 bound to ribosome color coded as in line diagram, with ZHD positioned at H24 and eL31. (B and C) Cells expressing Zuo1 variants with Bpa incorporated at indicated positions were exposed to UV light (+) or left unexposed (−). Cross-linking was analyzed by immunoblotting after SDS–PAGE using antibodies specific for (anti) Zuo1, NACα or NACβ, as indicated. Zuo1^Bpa^–NAC cross-link products are indicated with arrowheads, and non-cross-linked proteins and migration of molecular weight markers (kDa) by dashes. (**B**) Identification of Zuo1^Bpa^ variants that cross-link to NAC. Absence (Δ) or presence (WT) of gene encoding NACβ. (**C**) Inhibition of translation initiation: no treatment (−); auxin analog NAA to reduce eIF3B levels (eIF down arrow); glucose starvation (GS). (**D**) Cartoon representation of Zuo1 residues 75–285. Positions that cross-link to NAC when Bpa is incorporated are represented as spheres. HPD of JD and arginine residues known to be critical for ribosome association via interaction with H24 shown in stick representation.

To address whether Zuo1 and NAC can be on the ribosome simultaneously, we carried out a Bpa cross-linking screen of Zuo1, concentrating on segments of Zuo1 that, based on X-ray crystallography (16), computational modeling (34,35,39) and cryo-EM data (37,48), face toward the tunnel exit—a short segment just N-terminal to the J-domain, and portions of the ZHD (Figure 2A). We detected Zuo1^Bpa^–NAC cross-link products when Bpa was placed at (i) five positions just N-terminal to the J-domain, between D84 and D92; (ii) two positions in the loop prior to ZHD’s helix III (E238 and D239); and (iii) nine positions in helix III itself, between K250 and D268 (Figure 2 and Supplementary Figure S2). Several Bpa variants yielded cross-links to both the NACα subunit and the NACβ subunit, while some positions in ZHD helix III (e.g. K256Bpa) cross-linked to NACα, but not NACβ.

The Zuo1^Bpa^–NAC cross-links observed could be either to NAC that is interacting directly with the ribosome or to NAC that is no longer binding the ribosome directly, but present in the ribosomal pellet because of interaction with nascent polypeptides. To discriminate between these possibilities, we assessed cross-linking of Zuo1 variants having Bpa at two positions—R91 and K250—under conditions that inhibit translation initiation, but not elongation, thus allowing runoff of ribosomes from mRNA. Two approaches were used: short-term (10 min) glucose starvation (49,50) and depletion of initiation factor eIF3b using an auxin-inducible degron (51). Both treatments resulted in substantial reduction in polysomes and reduced cross-linking of Ssb^Bpa^ to ribosomal proteins uL24 and uL29 across the tunnel, as expected upon severe reduction in the number of nascent chains (19) (Supplementary Figure S3). Under both conditions, substantial cross-linking between Zuo1^Bpa^ and NAC still occurred (Figure 2C). We interpret these results as indicating that Zuo1 and NAC can be on a nontranslating ribosome at the same time.

### Helical region of the NAC globular domain cross-links to Zuo1 *in vivo*

**Figure 3.**
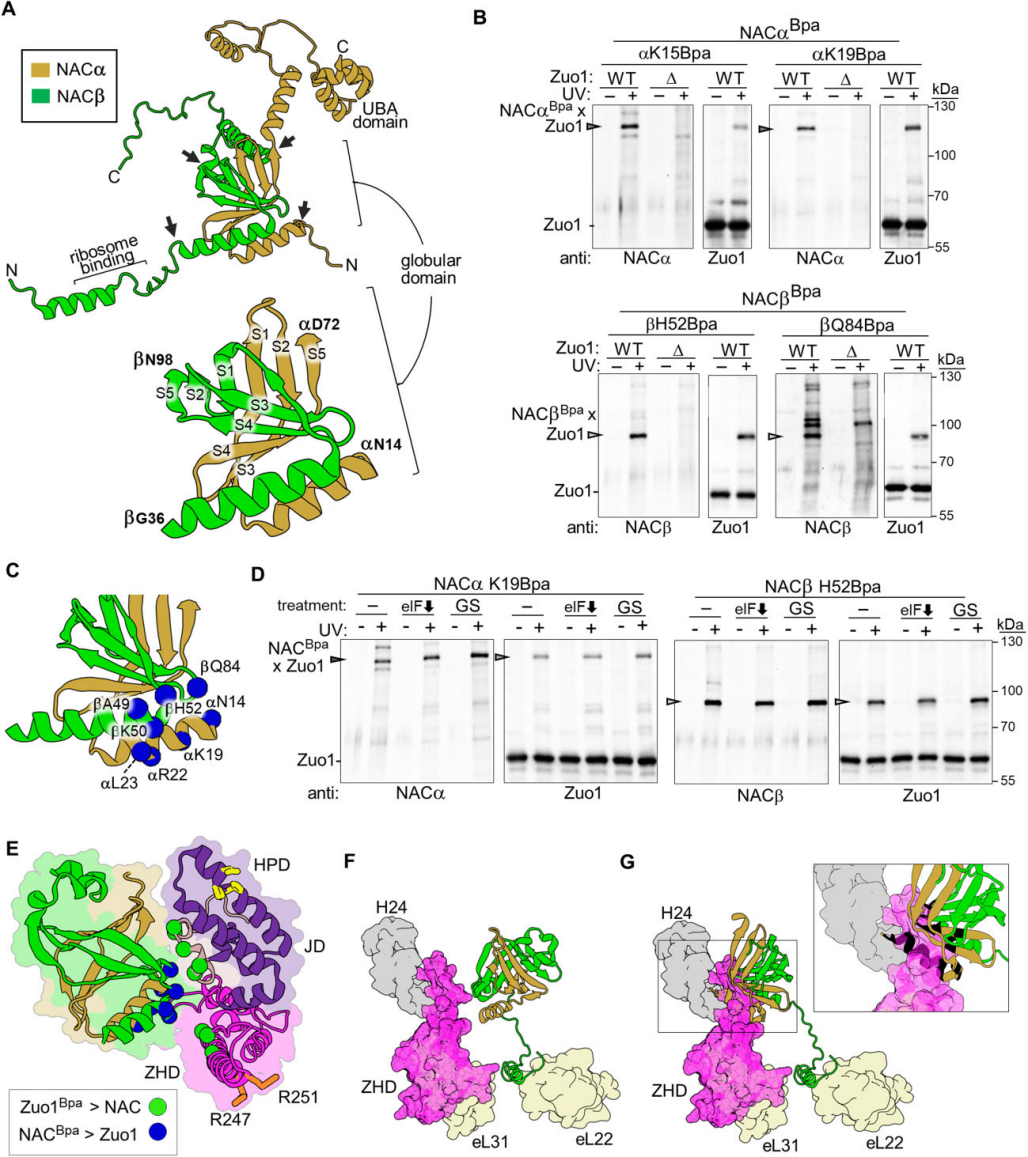
NAC globular domain cross-links to Zuo1 *in vivo*. (**A**) Model of NAC heterodimer (DOI: 10.5452/ma-bak-cepc-0495). Bracket designates the globular domain into which Bpa was incorporated, with the beginning and end of domain indicated by arrows and residue for each subunit indicated; expanded view of globular domain with β-strands designated (S1, S2, etc.) below. (B and C) Cells expressing NACα and NACβ variants with Bpa incorporated at indicated positions were exposed to UV light (+) or left unexposed (−). Cross-linking was analyzed by immunoblotting after SDS–PAGE using antibodies specific for (anti) NACα, NACβ or Zuo1. NAC^Bpa^–Zuo1 cross-link products indicated with arrowheads, and non-cross-linked proteins and migration of molecular weight markers (kDa) by dashes; (**B**) absence (Δ) or presence (WT) of gene encoding Zuo1; (**C**) NAC globular domain with positions of Bpa incorporation giving cross-links to Zuo1 represented as spheres. (**D**) Inhibition of translation initiation: no treatment (−); NAA to reduce eIF3b (eIF down arrow); glucose starvation (GS). (**E**) Positioning of NAC and Zuo1. Based on the Zuo1 Bpa positions that cross-linked to both NACα and NACβ (Figure 2) and NACα and NACβ Bpa positions that cross-linked to Zuo1 as in panel (D), as well as the established interaction of NACβ N-terminus with the ribosome; NAC was positioned relative to Zuo1 and then refined using PyRosetta (40). Positive Bpa positions shown as spheres. Only Zuo1 positions that cross-link to both NACα and NACβ are shown. (F and G) Comparison of models of NAC positioning in the presence and absence of Zuo1 with the N-terminus of NACβ included. For clarity, only the ZHD of Zuo1 is shown. (**F**) Expanded view of positioning in panel (E), rotated 90 degrees. (**G**) Position of NAC at the ribosome in the absence of Zuo1. Mammalian cryo-EM structure of RNC–NAC complex (10) (PDBID: 7QWR) was superimposed on the ribosome–Zuo1 model to illustrate incompatibility of the coexistence of Zuo1 and NAC in this orientation. Upper right: box indicates zoomed in segment showing region of clash between Zuo1 and NAC. Transparency of Zuo1 increased for clarity with NAC residues in conflict indicated in black.

To probe the relative positioning of Zuo1 and NAC at the ribosome, we incorporated Bpa into NAC. Because a number of Zuo1^Bpa^ positions cross-linked to both the α and the β subunit, we reasoned that cross-linking of Zuo1^Bpa^ was likely occurring to the globular domain that is formed via extended contacts between the two subunits (Figure 3A). We scanned this domain, incorporating Bpa into 41 positions—20 in NACα and 21 in NACβ. The strongest cross-links were detected when Bpa was incorporated in NACα at position αK19, and in NACβ at positions βH52 and βQ84 (Figure 3B). Though in different polypeptide chains, these Bpa positions are in close proximity in the NAC heterodimer—in the N-terminal segment of the NACα helix, and near the C-terminal end of the NACβ helix (βH52), as well as in the nearby loop between β-strands 3 and 4 (βQ84). Other Bpa positions for which Zuo1 cross-link products were detected lie along either the α or the β helix (Figure 3C and Supplementary Figure S4). NAC^Bpa^–Zuo1 cross-linking was similar whether translation initiation was inhibited or not (Figure 3D)—indicating that NAC^Bpa^ –Zuo1 cross-linking, like Zuo1^Bpa^–NAC cross-linking, can occur on nontranslating ribosomes.

While the data presented in this (Figure 3) and the previous (Figure 2) section are not sufficient to precisely position NAC relative to Zuo1 on the ribosome, they provide constraints—the face of the globular domain formed by one end of the antiparallel helices is in close proximity to the N-terminal half of helix III of the ZHD, the portion of the loop immediately above it and a region immediately proximal to the J-domain (Figure 3E). In such a placement, the N-terminus of the NACβ helix is down toward, and that of NACα up away from, the ribosome surface (Figure 3F). Notably, it is possible to accommodate these cross-linking data with positioning of the positively charged residues of NACβ’s N-terminal extension from the globular domain that is important for NAC–ribosome binding (8,9)—near eL22 and eL19, as shown for human NAC–ribosome complexes by cryo-EM analysis (Figure 3G) (10).

However, the Zuo1–NAC cross-linking results are not compatible with the cryo-EM-derived placement of the globular domain of NAC (10)—close to the tip of H24, with the NACα helix abutting it (Figure 3G). Such positioning would preclude ribosome binding of Zuo1, as the interaction of H24 with the N-terminal arginines of helix III of Zuo1’s ZHD is critical for its association with the ribosome (16). Together, these results suggest that the globular domain positions differently depending on whether Zuotin is present. The NAC positioning we deduce from cross-linking in the presence of Zuo1 and that observed in the cryo-EM analysis are similar in the sense that the antiparallel helices of the globular domain face in the general direction of H24 (Figure 3F). This similarity makes it quite straightforward to envision the positioning we deduced in the case of joint occupancy transitioning to that observed when Zuotin is not present—that is, in the less complex *in vitro* system or *in vivo* where the abundance of Zuo1 is one-third to one half that of NAC. However, it should be kept in mind that being able to accommodate does not necessarily mean the same affinity for the ribosome when both, rather than only one, are present. Indeed, previous competition experiments indicated that addition of excess NAC to cell extracts competes ribosome-bound *Caenorhabditis elegans* Zuotin away from the ribosome (23).

### βSBD of Hsp70 Ssb1 cross-links to NAC

**Figure 4.**
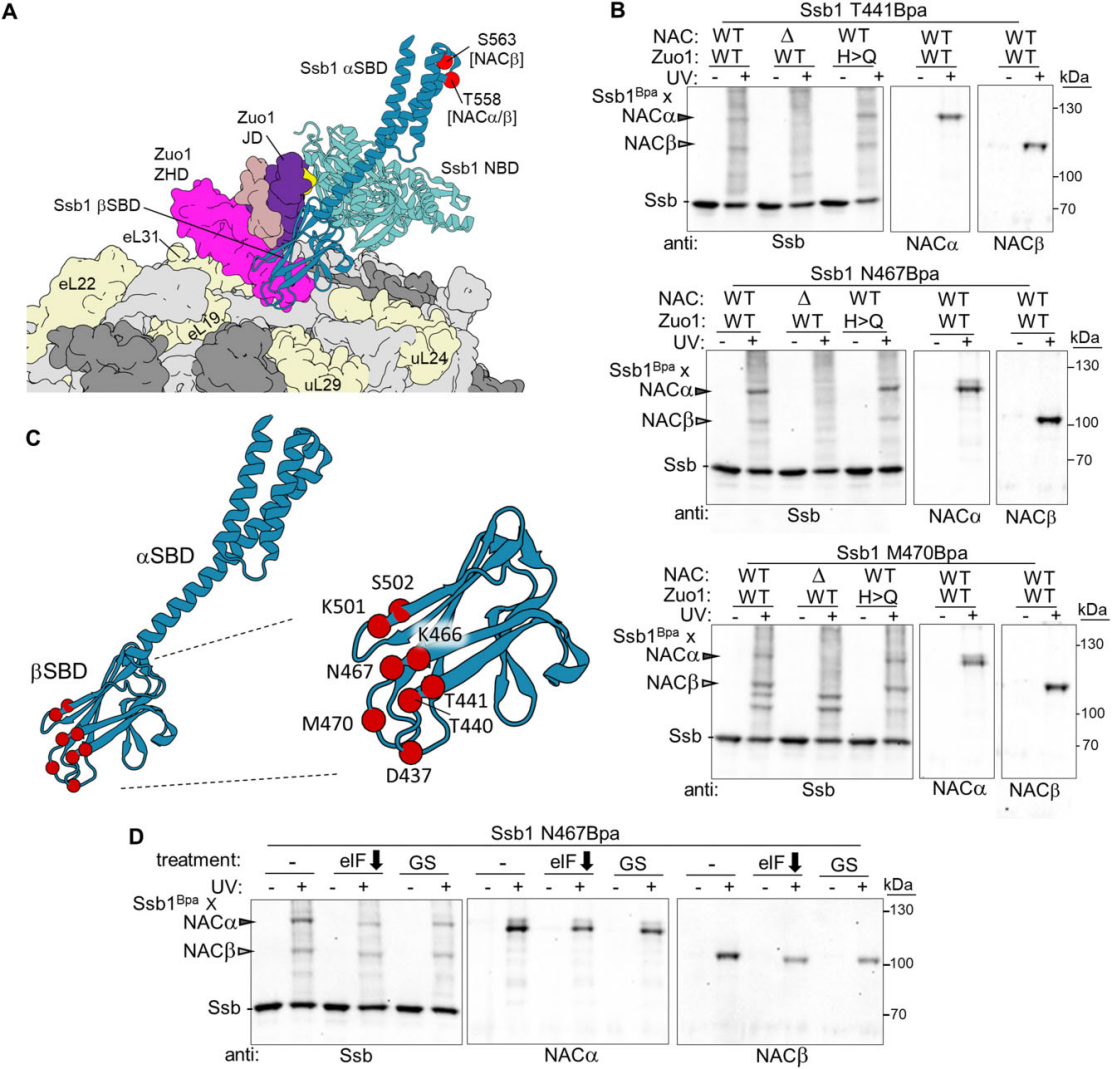
Both αSBD and βSBD of Ssb1 cross-link to NAC subunits. (**A**) Model of Ssb1(ATP) at the ribosome based on its interaction with the J-domain of Zuo1. Ssb1 in cartoon representation. Positions in αSBD that cross-link to NAC as in Figure 1 shown as spheres. Ribosome and Zuo1 (residues 75–285) shown in surface representation. Ribosome: ribosomal proteins discussed in manuscript in are labeled, while rRNA and other ribosomal proteins are light and dark gray, respectively. The Ssb1–Zuo1 complex was predicted using ColabFold (v1.5.2) implementation (43) of AlphaFold-Multimer-v3 (44) and positioned on the ribosome by fitting the Zuo1 and ribosome structure (PDBID: 6TNU) to cryo-EM map of RNC complex in complex with Zuo1 and Ssz1 (EMDB: EMD-32977). (B and D) Cells expressing Ssb1 variants with Bpa incorporated at indicated positions were exposed to UV light (+) or left unexposed (−). Cross-linking was analyzed by immunoblotting after SDS–PAGE using antibodies specific for (anti) Ssb, NACα or NACβ. Ssb1^Bpa^–NAC cross-link products are indicated with arrowheads, and non-cross-linked proteins and migration of molecular weight markers (kDa) by dashes. (**B**) Δ, deletion of gene encoding NACβ; H>Q, H128Q substitution in Zuo1 J-domain. (**C**) Left: Ssb1 SBD with positions in βSBD that cross-link to NAC indicated by spheres; right: further enlarged βSBD with positions labeled. (**D**) Ssb1^N467Bpa^ cross-linking after inhibition of translation initiation: no treatment (−); NAA treatment to reduce eIF3b (eIF down arrow); glucose starvation (GS).

Our initial cross-linking (Figure 1) indicated that the αSBD lid of Ssb(ATP) is in very close proximity to NAC. To understand the relative positioning of the NAC globular domain and Ssb(ATP), we made use of the known interaction of J-domains and the ATP conformation of Hsp70s—at the interface between the NBD, the docked βSBD subdomain and interdomain linker (52,53) (Figure 1B). When we positioned Ssb1(ATP) at the ribosome relative to ribosome-bound Zuo1 based on this J-domain interaction (52,53), two features were immediately apparent (Figure 4A): (i) the lid is far from the ribosome surface and (ii) βSBD having the peptide binding site is situated near the tunnel exit, in close proximity to both a nascent polypeptide chain emerging from the ribosome tunnel and, based on the Zuo1^Bpa^–NAC crossing results described above, the NAC globular domain (Figure 2). We therefore incorporated Bpa into Ssb1 βSBD positions predicted by the model to be closest to the NAC globular domain—the loops between the β-strands on the side of βSBD opposite from those that dock on the NBD. Cross-links to both NACα and NACβ were detected (Figure 4B and C, and Supplementary Figure S5). Substantial cross-linking also occurred in the *zuo1^H128Q^* background and after inhibition of translation initiation (Figure 4B and D), consistent with the cross-links occurring between Ssb1(ATP)^Bpa^ and NAC even in the absence of nascent chains.

### Positioning of NAC relative to Zuo1 and βSBD of Ssb

**Figure 5.**
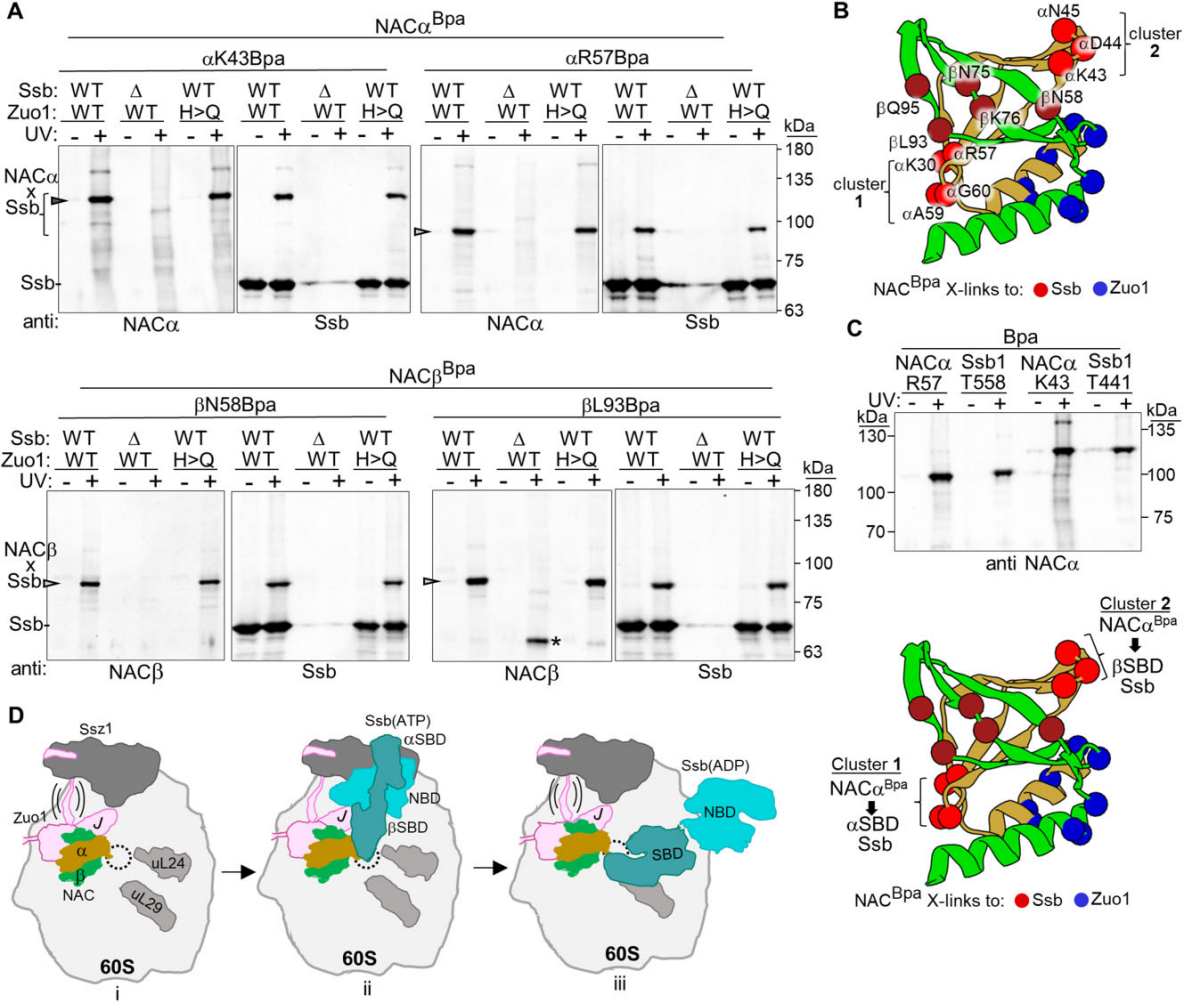
Juxtaposition of NAC and Zuo1/Ssb at the ribosome. (A and C) Cells expressing NACα, NACβ or Ssb1 variants with Bpa incorporated at indicated positions were exposed to UV light (+) or left unexposed (−). Cross-linking was analyzed by immunoblotting after SDS–PAGE using antibodies specific for Ssb, NACα or NACβ, as indicated (anti). Cross-link products are indicated with arrowheads, and migration of molecular weight markers (kDa) by dashes. (**A**) Bpa incorporated into NACα or NACβ. Δ, *SSB1 SSB2* deletion; H>Q, H128Q substitution in Zuo1 J-domain; WT, wild-type *SSB1*, *SSB2* and *ZUO1*. Asterisk (*) indicates cross-link of NACβ^Bpa^ to an unidentified protein that increases when Ssb is absent. (**B**) NAC globular domain with NACα or NACβ positions forming cross-links represented as spheres: to Zuo1 (as shown in Figure 3); to Ssb (as shown in A and Supplementary Figure S6). (**C**) (Top) Side by side comparison of migration of NACα^Bpa^ and Ssb1^Bpa^ cross-link products. Two prestained markers were used as discussed in the ‘Materials and methods’ section. (Bottom) NAC globular domain as in panel (B), with putative identification of NACα cross-links to Hsp70 αSBD and βSBD subdomains deduced from migration of cross-link products indicated by brackets. (**D**) Model of co-occupancy of Zuo1/NAC/Ssb on the 60S ribosomal subunit. (i) Joint occupancy of Zuo1 and NAC. Though capable of binding independently, Zuo1/Ssz1 (RAC) and NAC can occupy the ribosome at the same time. *Note*: Only globular domain of NAC is depicted. Brackets indicate the flexibility of the N-terminal segment of Zuo1, which binds tightly to the atypical Hsp70 Ssz1, tethering it to the ribosome. (ii) Positioning of Ssb(ATP). Ssb(ATP) interacts with Ssz1 and with Zuo1’s J-domain. *Note*: Loops of SBDβ near tunnel exit are in close proximity to NAC. (iii) After binding of nascent chain in the peptide binding cleft and hydrolysis of ATP stimulated by J-domain, the SBD of Ssb(ADP) binds directly to the ribosome interacting with uL24, uL29 and rRNA. *Note*: This positioning does not preclude binding of Zuo1 and/or NAC.

Next, to better understand the relative positioning of Ssb(ATP) and the NAC globular domain, the NACα and NACβ Bpa variants constructed for Zuo1 testing were screened for cross-linking to Ssb (Figure 5A and Supplementary Figure S6). Both NACα^Bpa^ and NACβ^Bpa^ cross-links to Ssb were detected. After testing of several additional globular domain NAC^Bpa^ variants, it was evident that positions cross-linking to Ssb were distinct from those cross-linking to Zuo1 (Figure 5B). Those that cross-linked to Ssb were predominantly in loops between the β-strands, rather than in or close to the helices. In addition, the positions cross-linking, based on the positioning of NAC shown in Figure 3, were mainly on a side of the globular domain that faces toward the tunnel exit, rather than toward the 40S subunit.

The NACα^Bpa^–Ssb cross-link products formed two spatial clusters—those in the loops between the helix and β-strand 1 (K30) and those between β-strands 1 and 2 (R57, A59, G70) forming one cluster and those in the loop between strands 3 and 4 (K43, R44, N45) forming a second (Figure 5B). We noted that the cross-link products from the two clusters migrated differently, with an apparent molecular weight difference of ∼30 kDa. When analyzing Ssb1^Bpa^–NACα cross-links, we had noted migration differences of a similar magnitude. The apparent molecular weight of cross-link products between the same two proteins may differ depending on what segments cross-link (54). We therefore compared on the same gel the relative migration of NACα^Bpa^–Ssb1 and Ssb1^Bpa^–NACα cross-link products. More specifically, Bpa was incorporated into Ssb1’s SBD subdomains (position 558 of αSBD or position 441 of βSBD) to those of representatives of NACα^BPA^ cluster 1 and cluster 2 (Figure 5C). Group 1 NACα^Bpa^ product migrated similarly to that of Ssb1 αSBD^Bpa^, and cluster 2 NACα^Bpa^ product similarly to that of Ssb1 βSBD^Bpa^.

These comparative results suggest that Bpa at cluster 2 positions of NACα cross-link to the Ssb1 βSBD subdomain (Figure 4C), compatible with Zuo1’s J-domain predicted interaction with Ssb1(ATP) at the ribosome (45,46,47). The cross-linking of Ssb1’s αSBD lid is more difficult to interpret, as in the predicted arrangement the lid is distant from both NAC and the ribosome. Several explanations are possible. First, some Hsp70s, when bound to ATP, are not in a stable docked state—with both the α and the β SBD subdomains docked on the NBD (55). Rather, αSBD docking is transient, allowing movement and transient interaction with βSBD. The proximity of the two clusters of NACα^Bpa^ to Ssb cross-linking is potentially compatible with this explanation. Second, recent cryo-EM results make it apparent that parts of the Zuo1–Ssz1 heterodimer are highly mobile (37,48), due mainly to the flexibility of Zuo1’s N-terminus (56,57). Ssz1 has been reported to interact with short nascent chains via its peptide binding cleft and ‘pass them off’ to Ssb1, with which it forms a heterodimer (57). Such maneuvering would result in a dramatic positional shift of an Ssz1–Ssb1 heterodimer. It is conceivable that such a shift could result in close proximity of the αSBD lid of Ssb to the NAC globular domain.

### Concluding remarks

Data presented here demonstrate that not only can Zuotin and NAC bind to the ribosome at the same time, but Ssb Hsp70 is able to position effectively near the exit even in the crowded environment created by NAC’s presence (Figure 5D). But how NAC and RAC coexistence on the ribosome is attained is not clear. Not only is the observed positioning of the NAC globular domain in conflict with Zuo1 ribosome binding, but NAC is more abundant than Zuo1. It is generally thought that RAC cycles on and off the ribosome (58), allowing Ssb’s known association with a wide variety of nascent chains (59,60). Further experiments are required to resolve this apparent conundrum, but several possibilities are evident. The positioning of the globular domain may well be more dynamic than available cryo-EM structures suggest. The N-terminus can position inside the tunnel when no nascent chain is present (23) or at the site discussed here, near eL19 and eL22 (10). Though the globular domain is positioned similarly in these structures, movement may occur, especially at the times of such switching or when interacting with specific nascent chains. Or, NAC may leave the ribosome altogether upon binding to nascent chains, allowing RAC free access prior to binding of another NAC molecule.

Regardless of mechanism, our results are consistent with the idea that the positioning of proteins involved in maturation and folding of nascent polypeptide chains near the tunnel exit site is adaptable—an idea gaining traction in regard to NAC and the less abundant, but critical, signal recognition particle (SRP) and methionine amino transferases (10,22,61). In the case of NAC and RAC/Hsp70 discussed here, their ability to occupy the same ribosome likely fosters more rapid, productive chaperone access to nascent chains. This could simply be because both systems can, simultaneously, be in close proximity to the nascent chain. However, the situation may be more nuanced than simple coexistence, as illustrated by the productive interplay at the exit tunnel of NAC with targeting signals for translocation into the endoplasmic reticulum and SRP (10,58,62). Whether NAC and the Hsp70 system, particularly Zuotin, are involved in such interplay remains an open question, as does the relative importance of NAC’s role in facilitating nascent chain folding compared to its emerging role as the ‘regulator’ of access of other factors to nascent chains. If the latter, then early access of the Hsp70 system to nascent chains may well be of prime importance for aggregation prevention and facilitation of folding, and may help explain why this system has evolved such complex architecture. Regardless, our results underscore the value of assessing interactions occurring in the crowded cellular milieu, along with informative structural studies carried out with purified components. These approaches are complementary and together can provide a fuller view of the complexity of protein folding/homeostasis systems.

## Supplementary Material

gkae005_Supplemental_File

## Data Availability

The mass spectrometry proteomics data underlying this article are available in the ProteomeXchange Consortium via the PRIDE partner repository with the dataset identifier 10.6019/PXD046895.

## References

[1] Balchin D. , Hayer-Hartl M., Hartl F.U. *In vivo* aspects of protein folding and quality control. Science. 2016; 353:aac4354. 27365453 10.1126/science.aac4354

[2] Kramer G. , Shiber A., Bukau B. Mechanisms of cotranslational maturation of newly synthesized proteins. Annu. Rev. Biochem. 2019; 88:337–364. 30508494 10.1146/annurev-biochem-013118-111717

[3] Deuerling E. , Gamerdinger M., Kreft S.G. Chaperone interactions at the ribosome. Cold Spring Harb. Perspect. Biol. 2019; 11:a033977. 30833456 10.1101/cshperspect.a033977 PMC6824243

[4] Zhang Y. , Sinning I., Rospert S. Two chaperones locked in an embrace: structure and function of the ribosome-associated complex RAC. Nat. Struct. Mol. Biol. 2017; 24:611–619. 28771464 10.1038/nsmb.3435

[5] Wiedmann B. , Sakai H., Davis T.A., Wiedmann M. A protein complex required for signal-sequence-specific sorting and translocation. Nature. 1994; 370:434–440. 8047162 10.1038/370434a0

[6] Liu Y. , Hu Y., Li X., Niu L., Teng M. The crystal structure of the human nascent polypeptide-associated complex domain reveals a nucleic acid-binding region on the NACA subunit. Biochemistry. 2010; 49:2890–2896. 20214399 10.1021/bi902050p

[7] Wang L. , Zhang W., Wang L., Zhang X.C., Li X., Rao Z. Crystal structures of NAC domains of human nascent polypeptide-associated complex (NAC) and its αNAC subunit. Protein Cell. 2010; 1:406–416. 21203952 10.1007/s13238-010-0049-3 PMC4875098

[8] Pech M. , Spreter T., Beckmann R., Beatrix B. Dual binding mode of the nascent polypeptide-associated complex reveals a novel universal adapter site on the ribosome. J. Biol. Chem. 2010; 285:19679–19687. 20410297 10.1074/jbc.M109.092536 PMC2885246

[9] Wegrzyn R.D. , Hofmann D., Merz F., Nikolay R., Rauch T., Graf C., Deuerling E. A conserved motif is prerequisite for the interaction of NAC with ribosomal protein L23 and nascent chains. J. Biol. Chem. 2006; 281:2847–2857. 16316984 10.1074/jbc.M511420200

[10] Jomaa A. , Gamerdinger M., Hsieh H.H., Wallisch A., Chandrasekaran V., Ulusoy Z., Scaiola A., Hegde R.S., Shan S.O., Ban N. et al. Mechanism of signal sequence handover from NAC to SRP on ribosomes during ER-protein targeting. Science. 2022; 375:839–844. 35201867 10.1126/science.abl6459 PMC7612438

[11] Mayer M.P. , Gierasch L.M. Recent advances in the structural and mechanistic aspects of Hsp70 molecular chaperones. J. Biol. Chem. 2019; 294:2085–2097. 30455352 10.1074/jbc.REV118.002810 PMC6369304

[12] Peisker K. , Chiabudini M., Rospert S. The ribosome-bound Hsp70 homolog Ssb of *Saccharomyces cerevisiae*. Biochim. Biophys. Acta. 2010; 1803:662–672. 20226819 10.1016/j.bbamcr.2010.03.005

[13] Pfund C. , Huang P., Lopez-Hoyo N., Craig E.A. Divergent functional properties of the ribosome-associated molecular chaperone Ssb compared with other Hsp70s. Mol. Biol. Cell. 2001; 12:3773–3782. 11739779 10.1091/mbc.12.12.3773 PMC60754

[14] Kampinga H.H. , Craig E.A. The HSP70 chaperone machinery: J proteins as drivers of functional specificity. Nat. Rev. Mol. Cell Biol. 2010; 11:579–592. 20651708 10.1038/nrm2941 PMC3003299

[15] Yan W. , Schilke B., Pfund C., Walter W., Kim S., Craig E.A. Zuotin, a ribosome-associated DnaJ molecular chaperone. EMBO J. 1998; 17:4809–4817. 9707440 10.1093/emboj/17.16.4809 PMC1170810

[16] Lee K. , Sharma R., Shrestha O.K., Bingman C.A., Craig E.A. Dual interaction of the Hsp70 J-protein cochaperone Zuotin with the 40S and 60S ribosomal subunits. Nat. Struct. Mol. Biol. 2016; 23:1003–1010. 27669034 10.1038/nsmb.3299 PMC5097012

[17] Kaschner L.A. , Sharma R., Shrestha O.K., Meyer A.E., Craig E.A. A conserved domain important for association of eukaryotic J-protein co-chaperones Jjj1 and Zuo1 with the ribosome. Biochim. Biophys. Acta. 2015; 1853:1035–1045. 25639645 10.1016/j.bbamcr.2015.01.014 PMC4380617

[18] Gautschi M. , Lilie H., Funfschilling U., Mun A., Ross S., Lithgow T., Rucknagel P., Rospert S. RAC, a stable ribosome-associated complex in yeast formed by the DnaK–DnaJ homologs Ssz1p and zuotin. Proc. Natl Acad. Sci. U.S.A. 2001; 98:3762–3767. 11274393 10.1073/pnas.071057198 PMC31126

[19] Lee K. , Ziegelhoffer T., Delewski W., Berger S.E., Sabat G., Craig E.A. Pathway of Hsp70 interactions at the ribosome. Nat. Commun. 2021; 12:5666. 34580293 10.1038/s41467-021-25930-8 PMC8476630

[20] Raue U. , Oellerer S., Rospert S. Association of protein biogenesis factors at the yeast ribosomal tunnel exit is affected by the translational status and nascent polypeptide sequence. J. Biol. Chem. 2007; 282:7809–7816. 17229726 10.1074/jbc.M611436200

[21] Ho B. , Baryshnikova A., Brown G.W. Unification of protein abundance datasets yields a quantitative *Saccharomyces cerevisiae* proteome. Cell Syst. 2018; 6:192–205. 29361465 10.1016/j.cels.2017.12.004

[22] Nyathi Y. , Pool M.R. Analysis of the interplay of protein biogenesis factors at the ribosome exit site reveals new role for NAC. J. Cell Biol. 2015; 210:287–301. 26195668 10.1083/jcb.201410086 PMC4508901

[23] Gamerdinger M. , Kobayashi K., Wallisch A., Kreft S.G., Sailer C., Schlomer R., Sachs N., Jomaa A., Stengel F., Ban N. et al. Early scanning of nascent polypeptides inside the ribosomal tunnel by NAC. Mol. Cell. 2019; 75:996–1006. 31377116 10.1016/j.molcel.2019.06.030

[24] Sherman F. , Fink G., Hicks J. *Methods in Yeast Genetics* . 1986; Cold Spring Harbor, NY Cold Spring Harbor Press.

[25] Chen D.C. , Yang B.C., Kuo T.T. One-step transformation of yeast in stationary phase. Curr. Genet. 1992; 21:83–84. 1735128 10.1007/BF00318659

[26] Higgins D.A. , Young M.K.M., Tremaine M., Sardi M., Fletcher J.M., Agnew M., Liu L., Dickinson Q., Peris D., Wrobel R.L. et al. Natural variation in the multidrug efflux pump SGE1 underlies ionic liquid tolerance in yeast. Genetics. 2018; 210:219–234. 30045857 10.1534/genetics.118.301161 PMC6116967

[27] Kuang M.C. , Kominek J., Alexander W.G., Cheng J.F., Wrobel R.L., Hittinger C.T. Repeated *cis*-regulatory tuning of a metabolic bottleneck gene during evolution. Mol. Biol. Evol. 2018; 35:1968–1981. 29788479 10.1093/molbev/msy102 PMC6063270

[28] Morawska M. , Ulrich H.D. An expanded tool kit for the auxin-inducible degron system in budding yeast. Yeast. 2013; 30:341–351. 23836714 10.1002/yea.2967 PMC4171812

[29] Mumberg D. , Muller R., Funk M. Yeast vectors for the controlled expression of heterologous proteins in different genetic backgrounds. Gene. 1995; 156:119–122. 7737504 10.1016/0378-1119(95)00037-7

[30] Zhang Y. , Wolfle T., Rospert S. Interaction of nascent chains with the ribosomal tunnel proteins Rpl4, Rpl17, and Rpl39 of *Saccharomyces cerevisiae*. J. Biol. Chem. 2013; 288:33697–33707. 24072706 10.1074/jbc.M113.508283 PMC3837115

[31] Eisenman H.C. , Craig E.A. Activation of pleiotropic drug resistance by the J-protein and Hsp70-related proteins, Zuo1 and Ssz1. Mol. Microbiol. 2004; 53:335–344. 15225326 10.1111/j.1365-2958.2004.04134.x

[32] Lopez-Buesa P. , Pfund C., Craig E.A. The biochemical properties of the ATPase activity of a 70-kDa heat shock protein (Hsp70) are governed by the C-terminal domains. Proc. Natl Acad. Sci. U.S.A. 1998; 95:15253–15258. 9860955 10.1073/pnas.95.26.15253 PMC28029

[33] Sahi C. , Kominek J., Ziegelhoffer T., Yu H.Y., Baranowski M., Marszalek J., Craig E.A. Sequential duplications of an ancient member of the DnaJ-family expanded the functional chaperone network in the eukaryotic cytosol. Mol. Biol. Evol. 2013; 30:985–998. 23329686 10.1093/molbev/mst008 PMC3670730

[34] Jumper J. , Evans R., Pritzel A., Green T., Figurnov M., Ronneberger O., Tunyasuvunakool K., Bates R., Zidek A., Potapenko A. et al. Highly accurate protein structure prediction with AlphaFold. Nature. 2021; 596:583–589. 34265844 10.1038/s41586-021-03819-2 PMC8371605

[35] Varadi M. , Anyango S., Deshpande M., Nair S., Natassia C., Yordanova G., Yuan D., Stroe O., Wood G., Laydon A. et al. AlphaFold Protein Structure Database: massively expanding the structural coverage of protein-sequence space with high-accuracy models. Nucleic Acids Res. 2022; 50:D439–D444. 34791371 10.1093/nar/gkab1061 PMC8728224

[36] Buschauer R. , Matsuo Y., Sugiyama T., Chen Y.H., Alhusaini N., Sweet T., Ikeuchi K., Cheng J., Matsuki Y., Nobuta R. et al. The Ccr4–Not complex monitors the translating ribosome for codon optimality. Science. 2020; 368:eaay6912. 32299921 10.1126/science.aay6912 PMC8663607

[37] Chen Y. , Tsai B., Li N., Gao N. Structural remodeling of ribosome associated Hsp40–Hsp70 chaperones during co-translational folding. Nat. Commun. 2022; 13:3410. 35701497 10.1038/s41467-022-31127-4 PMC9197937

[38] Pettersen E.F. , Goddard T.D., Huang C.C., Meng E.C., Couch G.S., Croll T.I., Morris J.H., Ferrin T.E. UCSF ChimeraX: structure visualization for researchers, educators, and developers. Protein Sci. 2021; 30:70–82. 32881101 10.1002/pro.3943 PMC7737788

[39] Humphreys I.R. , Pei J., Baek M., Krishnakumar A., Anishchenko I., Ovchinnikov S., Zhang J., Ness T.J., Banjade S., Bagde S.R. et al. Computed structures of core eukaryotic protein complexes. Science. 2021; 374:eabm4805. 34762488 10.1126/science.abm4805 PMC7612107

[40] Chaudhury S. , Lyskov S., Gray J.J. PyRosetta: a script-based interface for implementing molecular modeling algorithms using Rosetta. Bioinformatics. 2010; 26:689–691. 20061306 10.1093/bioinformatics/btq007 PMC2828115

[41] Gray J.J. , Moughon S., Wang C., Schueler-Furman O., Kuhlman B., Rohl C.A., Baker D Protein–protein docking with simultaneous optimization of rigid-body displacement and side-chain conformations. J. Mol. Biol. 2003; 331:281–299. 12875852 10.1016/s0022-2836(03)00670-3

[42] Forne I. , Ludwigsen J., Imhof A., Becker P.B., Mueller-Planitz F. Probing the conformation of the ISWI ATPase domain with genetically encoded photoreactive crosslinkers and mass spectrometry. Mol. Cell. Proteomics. 2012; 11:M111.012088. 10.1074/mcp.M111.012088 PMC3322568 22167269

[43] Mirdita M. , Schutze K., Moriwaki Y., Heo L., Ovchinnikov S., Steinegger M. ColabFold: making protein folding accessible to all. Nat. Methods. 2022; 19:679–682. 35637307 10.1038/s41592-022-01488-1 PMC9184281

[44] Evans R. , O’Neill M., Pritzel A., Antropova N., Senior A., Green T., Žídek A., Bates R., Blackwell S., Yim J. et al. Protein complex prediction with AlphaFold-Multimer. 2022; bioRxiv doi:10 March 2022, preprint: not peer reviewed 10.1101/2021.10.04.463034.

[45] Hanebuth M.A. , Kityk R., Fries S.J., Jain A., Kriel A., Albanese V., Frickey T., Peter C., Mayer M.P., Frydman J. et al. Multivalent contacts of the Hsp70 Ssb contribute to its architecture on ribosomes and nascent chain interaction. Nat. Commun. 2016; 7:13695. 27917864 10.1038/ncomms13695 PMC5150220

[46] Gumiero A. , Conz C., Gese G.V., Zhang Y., Weyer F.A., Lapouge K., Kappes J., von Plehwe U., Schermann G., Fitzke E. et al. Interaction of the cotranslational Hsp70 Ssb with ribosomal proteins and rRNA depends on its lid domain. Nat. Commun. 2016; 7:13563. 27882919 10.1038/ncomms13563 PMC5123055

[47] Huang P. , Gautschi M., Walter W., Rospert S., Craig E.A. The Hsp70 Ssz1 modulates the function of the ribosome-associated J-protein Zuo1. Nat. Struct. Mol. Biol. 2005; 12:497–504. 15908962 10.1038/nsmb942

[48] Kisonaite M. , Wild K., Lapouge K., Gese G.V., Kellner N., Hurt E., Sinning I. Structural inventory of cotranslational protein folding by the eukaryotic RAC complex. Nat. Struct. Mol. Biol. 2023; 30:670–677. 37081320 10.1038/s41594-023-00973-1 PMC10191838

[49] Ashe M.P. , De Long S.K., Sachs A.B. Glucose depletion rapidly inhibits translation initiation in yeast. Mol. Biol. Cell. 2000; 11:833–848. 10712503 10.1091/mbc.11.3.833 PMC14814

[50] Bresson S. , Shchepachev V., Spanos C., Turowski T.W., Rappsilber J., Tollervey D Stress-induced translation inhibition through rapid displacement of scanning initiation factors. Mol. Cell. 2020; 80:470–484. 33053322 10.1016/j.molcel.2020.09.021 PMC7657445

[51] Papagiannakis A. , de Jonge J.J., Zhang Z., Heinemann M. Quantitative characterization of the auxin-inducible degron: a guide for dynamic protein depletion in single yeast cells. Sci. Rep. 2017; 7:4704. 28680098 10.1038/s41598-017-04791-6 PMC5498663

[52] Kityk R. , Kopp J., Mayer M.P. Molecular mechanism of J-domain-triggered ATP hydrolysis by Hsp70 chaperones. Mol. Cell. 2018; 69:227–237. 29290615 10.1016/j.molcel.2017.12.003

[53] Tomiczek B. , Delewski W., Nierzwicki L., Stolarska M., Grochowina I., Schilke B., Dutkiewicz R., Uzarska M.A., Ciesielski S.J., Czub J. et al. Two-step mechanism of J-domain action in driving Hsp70 function. PLoS Comput. Biol. 2020; 16:e1007913. 32479549 10.1371/journal.pcbi.1007913 PMC7289447

[54] Ting S.Y. , Yan N.L., Schilke B.A., Craig E.A. Dual interaction of scaffold protein Tim44 of mitochondrial import motor with channel-forming translocase subunit Tim23. eLife. 2017; 6:e23609. 28440746 10.7554/eLife.23609 PMC5422074

[55] Clerico E.M. , Meng W., Pozhidaeva A., Bhasne K., Petridis C., Gierasch L.M. Hsp70 molecular chaperones: multifunctional allosteric holding and unfolding machines. Biochem. J. 2019; 476:1653–1677. 31201219 10.1042/BCJ20170380 PMC7219557

[56] Weyer F.A. , Gumiero A., Gese G.V., Lapouge K., Sinning I. Structural insights into a unique Hsp70–Hsp40 interaction in the eukaryotic ribosome-associated complex. Nat. Struct. Mol. Biol. 2017; 24:144–151. 28067917 10.1038/nsmb.3349

[57] Zhang Y. , Valentin Gese G., Conz C., Lapouge K., Kopp J., Wolfle T., Rospert S., Sinning I. The ribosome-associated complex RAC serves in a relay that directs nascent chains to Ssb. Nat. Commun. 2020; 11:1504. 32198371 10.1038/s41467-020-15313-w PMC7083937

[58] Gamerdinger M. , Deuerling E. Cotranslational sorting and processing of newly synthesized proteins in eukaryotes. Trends Biochem. Sci. 2023; 10.1016/j.tibs.2023.10.003. 37919225

[59] Doring K. , Ahmed N., Riemer T., Suresh H.G., Vainshtein Y., Habich M., Riemer J., Mayer M.P., O’Brien E.P., Kramer G. et al. Profiling Ssb-nascent chain interactions reveals principles of Hsp70-assisted folding. Cell. 2017; 170:298–311. 28708998 10.1016/j.cell.2017.06.038 PMC7343536

[60] Willmund F. , del Alamo M., Pechmann S., Chen T., Albanese V., Dammer E.B., Peng J., Frydman J. The cotranslational function of ribosome-associated Hsp70 in eukaryotic protein homeostasis. Cell. 2013; 152:196–209. 23332755 10.1016/j.cell.2012.12.001 PMC3553497

[61] Gamerdinger M. , Jia M., Schloemer R., Rabl L., Jaskolowski M., Khakzar K.M., Ulusoy Z., Wallisch A., Jomaa A., Hunaeus G. et al. NAC controls cotranslational N-terminal methionine excision in eukaryotes. Science. 2023; 380:1238–1243. 37347872 10.1126/science.adg3297

[62] Coelho J.P.L. , Shao S. A ‘NAC’ for targeting proteins to the ER. Trends Biochem. Sci. 2022; 47:730–731. 35501234 10.1016/j.tibs.2022.04.007

