# Supplementary material for: NAC and Zuotin/Hsp70 chaperone systems coexist at the ribosome tunnel exit in vivo
Source: Nucleic Acids Res. 2024 Jan 15;52(6):3346–57. doi: 10.1093/nar/gkae005 (PMC11014269; doi:10.1093/nar/gkae005)
Supplement: gkae005_Supplemental_File [file gkae005_supplemental_file.pdf]

**SUPPLEMENTARY DATA**

# NAC and Zuotin/Hsp70 chaperone systems coexist at the ribosome tunnel exit in vivo

Thomas Ziegelhoffer[1,†], Amit K. Verma[1,†], Wojciech Delewski[1,†], Brenda A. Schilke[1], Paige M. Hill[1], Marcin Pitek[2], Jaroslaw Marszalek[1,2] and Elizabeth A Craig[1,*]

[1]Department of Biochemistry, University of Wisconsin-Madison, Madison, Wisconsin 53726, United States of America

[2]Intercollegiate Faculty of Biotechnology, University of Gdansk and Medical University of Gdansk, Gdansk 80-307, Poland

[†]these authors contributed equally to this work

*to whom correspondence should be addressed. ecraig@wisc.edu

**Supplementary Table 1.**
**Yeast strains used in this study.**

| Strain[1] | Genotype[2,3] | | Reference |
|---|---|---|---|
| Y1011 | *Δssb1::HIS3 Δssb2::LEU2* | | 1 |
| Y2907 | *Δssb1::HIS3 Δssb2::LEU2 Z:TAG>TAA* | | This study |
| Y2901 | *Δzuo1::HIS3 Z:TAG>TAA* | | This study |
| Y2934 | *Δzuo1::HIS3 Δssb1::HIS3 Δssb2::LEU2 Z:TAG>TAA* | | This study |
| Y2904 | *Δedg1::KanMX Z:TAG>TAA* | | This study |
| Y2906 | *Δedg1 Δzuo1::HIS3 Z:TAG>TAA* | | This study |
| Y2881 | *Δegd1::Nat Δssb1::HIS3 Δssb2::KanMX* | | This study |
| Y2946 | *Δegd1 Δssb1::HIS3 Δssb2::LEU2 Z:TAG>TAA* | | This study |
| Y2905 | *Δegd2 Z:TAG>TAA* | | This study |
| Y2908 | *Δegd2 Δzuo1::HIS3 Z:TAG>TAA* | | This study |
| Y2947 | *Δegd2 Δssb1::HIS3 Δssb2::LEU2 Z:TAG>TAA* | | This study |
| Y2987 | *Δegd2 Δegd1 Δzuo1::HIS3 Z:TAG>TAA* | | This study |
| Y2945 | *Δedg1 Z:TAG>TAA HO::OsTIR1-KanMX PRT1-aid* | | This study |
| Y2955 | *Δedg2 Z:TAG>TAA HO::OsTIR1-KanMX PRT1-aid* | | This study |
| Y2948 | *Δssb1::HIS3 Δssb2::LEU2 Z:TAG>TAA HO::OsTIR1-KanMX PRT1-aid* | | This study |

[1]all are DS10 strain background - *GAL2 his3-11,15 leu2-3,112 lys1 lys2 Δtrp1 ura3-52*
[2]Z:TAG>TAA indicates alteration of the native TAG stop codon to TAA to prevent Bpa incorporation
[3]mutations with no additional designation of marker were created by Crispr.

**References.**

1. Pfund, C., Huang, P., Lopez-Hoyo, N. & Craig, E.A. Divergent functional properties of the ribosome-associated molecular chaperone Ssb compared with other Hsp70s. *Mol Biol Cell* **12**, 3773-82 (2001).

**Supplementary Table 2.**

**Plasmids used in this study.**

| Plasmid | Description | References |
|---|---|---|
| pRS316-SSB1 | pRS316 carrying *SSB1* under control of its native promoter; used as template to generate all Ssb1 variants used in this study unless specified otherwise | 1 |
| pRS315-ZUO1 | pRS315 carrying *ZUO1* under control of its native promoter | 2 |
| pRS315-zuo1H128Q | pRS315 carrying $zuo1^{H128Q}$ (HPD loss of function mutant) under control of its native promoter | this study |
| pRS316-ZUO1 | pRS316 carrying *ZUO1* under control of its native promoter | 2 |
| pRS317-ZUO1 | pRS317 carrying *ZUO1* under control of its native promoter | 3 |
| pRS317-zuo1H128Q | pRS317 carrying $zuo1^{H128Q}$ (HPD loss of function mutant) under control of its native promoter | 4 |
| ptRNA-Bpa | 2 micron plasmid encoding a variant tRNA synthetase and tRNACUA for Bpa incorporation; *TRP1* marker | 5 |
| pRS415-GPD-NACβ | pRS415 centromeric plasmid carrying *NACβ* under GPD promoter | this study |
| pRS416-GPD-NACα | pRS416 centromeric plasmid carrying *NACα* under GPD promoter | this study |

**References**


1. James, P., Pfund, C. & Craig, E.A. Functional specificity among Hsp70 molecular chaperones. *Science* **275**, 387-9 (1997).
2. Yan, W. et al. Zuotin, a ribosome-associated DnaJ molecular chaperone. *EMBO J* **17**, 4809-17 (1998).
3. Eisenman, H.C. & Craig, E.A. Activation of pleiotropic drug resistance by the J-protein and Hsp70-related proteins, Zuo1 and Ssz1. *Mol Microbiol* **53**, 335-44 (2004).
4. Lee, K. et al. Pathway of Hsp70 interactions at the ribosome. *Nat Commun* **12**, 5666 (2021).
5. Krishnamurthy, M. et al. Caught in the act: covalent cross-linking captures activator-coactivator interactions in vivo. *ACS Chem Biol* **6**, 1321-6 (2011).

**Supplementary Table 3.**

**Mass Spectrometry of Ssb1$^{Bpa}$ crosslinking samples.**

**S563Bpa**

| protein | MW (kDa) | spectral count[1] -UV[2] | +UV |
|---|---|---|---|
| Ssb1 | 67 | 24 | 121 |
| NACβ | 17 | 0 | 18 |

**R545Bpa**

| protein | MW (kDa) | spectral count[1] -UV[2] | +UV |
|---|---|---|---|
| Ssb1 | 67 | 25 | 435 |
| uL29 | 14 | 3 | 46 |

[1] spectral count of peptides representing indicated proteins. Only those having 2-fold or greater value in +UV vs. untreated control are shown.

[2] gel slice of sample not UV treated from same position as crosslink product in +UV sample used as a control.

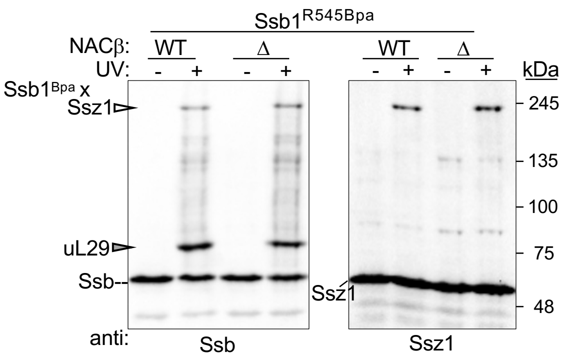


**Figure S1.** Crosslinking of Ssb1$^{R545Bpa}$ to Ssz1 in the absence of NACβ. Cells having or lacking the *EGD1* gene that encodes the predominant NACβ subunit variant and expressing Ssb1$^{R545Bpa}$ were exposed to UV light (+) or left unexposed (-). Crosslinking was analyzed by immunoblotting after SDS-PAGE using antibodies specific for (anti) Ssb and Ssz1, as indicated. Ssb1$^{Bpa}$ crosslink products are indicated with arrowheads; noncrosslinked proteins and migration of molecular weight markers (kDa) by dashes. Δ, deletion of *EGD1* gene encoding NACβ.

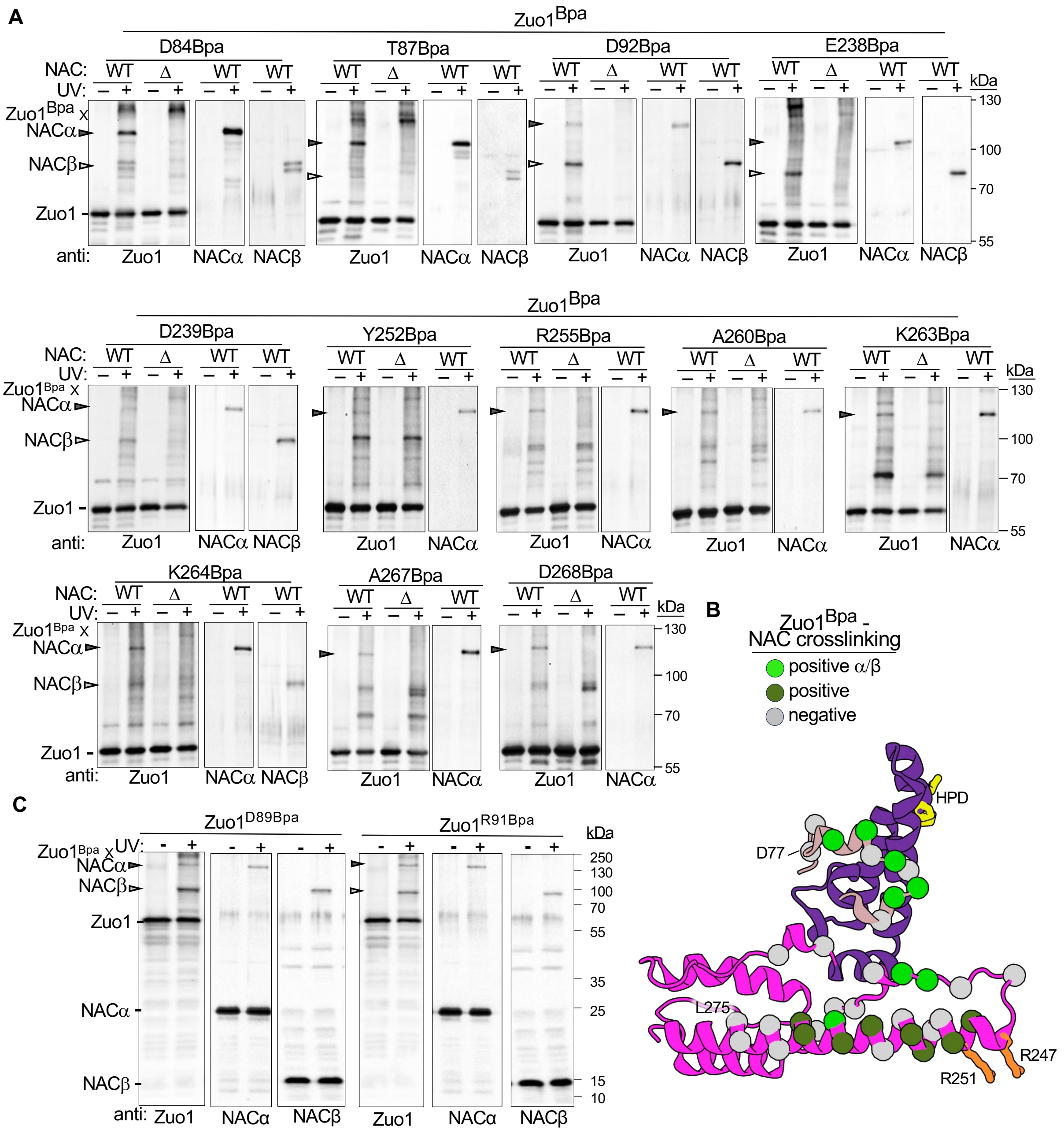


**Figure S2.** Crosslinking of Zuo1$^{\text{Bpa}}$ to NAC.

(A) Cells expressing Zuo1 variants with Bpa incorporated at indicated positions were exposed to UV light (+) or left unexposed (-). Crosslinking was analyzed by immunoblotting after SDS-PAGE using antibodies specific for (anti) Zuo1, NACα or NACβ, as indicated. Zuo1$^{\text{Bpa}}$-NAC crosslink products are indicated with arrowheads; noncrosslinked protein and migration of molecular weight markers (kDa) indicated by dashes. WT, wild-type NAC; Δ, deletion of *EGD1* gene encoding NACβ. (B) AlphaFold modeled structure of Zuo1 residues 75-285 (AF-A0A815YXL5). HPD (residues 128-130) of J-domain in yellow; arginine residues (R247, R251) known to be critical for ribosome association via interaction with H24 in orange. Positions having Bpa incorporated shown as spheres; crosslinks to NAC: positive, green (to both NACα and NACβ, bright green; NACα only, dark green); negative, light gray. Zuo1$^{\text{Bpa}}$ negatives and positives (in bold) are D77, E79, **D84**, **T87**, H88, **D89**, A90, **R91, D92,** K94, D168, D172, S229, R231, T232, L236, **E238**, **D239**, P241, S244, **K250**, **Y252**, I253, E254, **R255**, **K256**, N257, A259, **A260**, **K263**, **K264**, **A267**, **D268**, A270, R271, K274, L275. For clarity of distribution of Bpa positions only D77 and L275 are designated. See Figure 2D for labeling of positives. (C) samples of Zuo1D89Bpa and Zuo1R91Bpa were run on 13% gels to visualize NAC monomers.

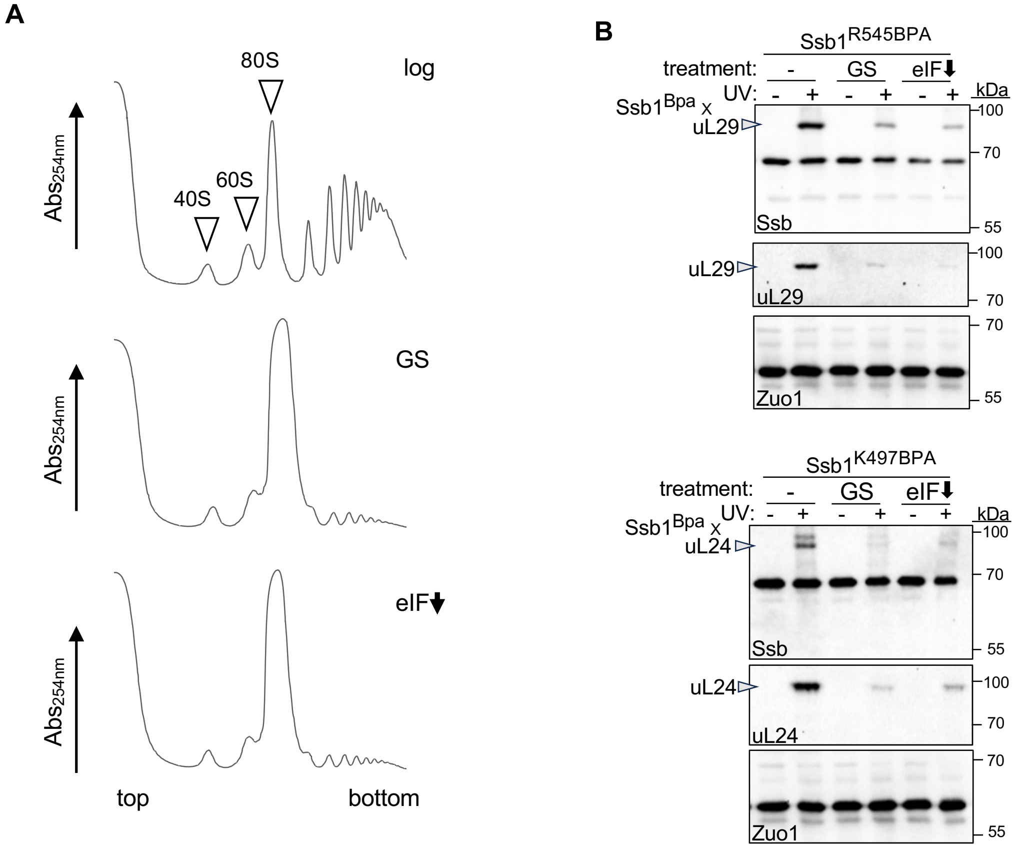


**Figure S3.** Inhibition of translation initiation.
(A) Polysome analysis of extracts of cells in log phase (log), after 10 min glucose starvation (GS) or auxin-induced depletion of eIF3B by treatment with the auxin analog 1-naphthaleneacetic acid (NAA) (eIF down arrow). Relative $Abs_{254nm}$ level was monitored over time as gradients were sampled continuously from top to bottom. Positions of 40S and 60S subunits are indicated, along with intact 80S ribosomes. (B) Crosslinking control after inhibition of translation initiation. Cells expressing Ssb1 variants with Bpa incorporated at indicated positions were exposed to UV light (+) or left unexposed (-). Crosslinking was analyzed by immunoblotting after SDS-PAGE using antibodies specific for Ssb, ribosomal proteins uL29 or uL24, and as a control Zuo1 - as indicated at bottom left of each panel. $Ssb1^{Bpa}$ crosslinks to ribosomal proteins are indicated by arrowheads; migration positions of molecular weight markers (kDa) by dashes.

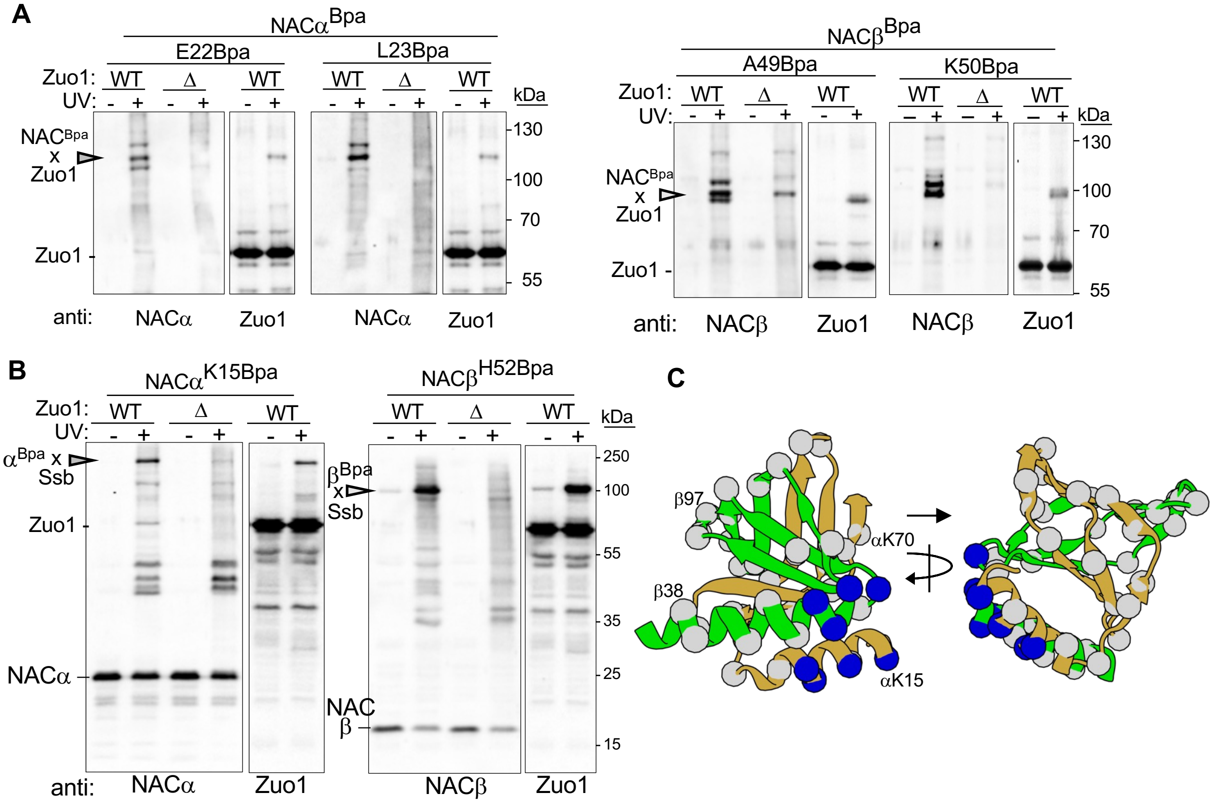


**Figure S4**. Crosslinking of NACα$^{Bpa}$ and NACβ$^{Bpa}$ to Zuo1.

(A, B) Cells expressing NACα and NACβ variants with Bpa incorporated at indicated positions were exposed to UV light (+) or left unexposed (-). Crosslinking was analyzed by immunoblotting after SDS-PAGE using antibodies specific for (anti) NACα, NACβ or Zuo1 as indicated. NAC$^{Bpa}$-Zuo1 crosslink products are indicated with arrowheads; noncrosslinked proteins and migration of molecular weight markers (kDa) indicated by dashes. Δ, deletion of gene encoding Zuo1. Samples in (B) were run on 13% gels to visualize NAC monomers. (C) Model of NAC globular domain (10.5452/ma-bak-cepc-0495); α subunit, brown; β subunit, green. Positions into which Bpa was incorporated and tested for crosslinking to Zuo1 are shown as spheres: positives, blue; negatives, light gray. NACα$^{Bpa}$ negatives and positives (bold): **K15**, K18, **K19**, **E22**, **L23**, G25, G28, Q31, I32, I35, R37, K43, N45, E51, E54, R57, A59, G67, A69, K70. NACβ$^{Bpa}$ negatives and positives (in bold): N38, K39, K43, Q45, Q47, **A49**, **K50**, **H52**, V54, E61, K66, D68, K70, H73, K76, Q80, **Q84**, H85, L93, E96, K97. For clarity, only αK15, αK70, β38 and β97 Bpa positions are designated on figure; see Figure 3D for labeling of all positives.

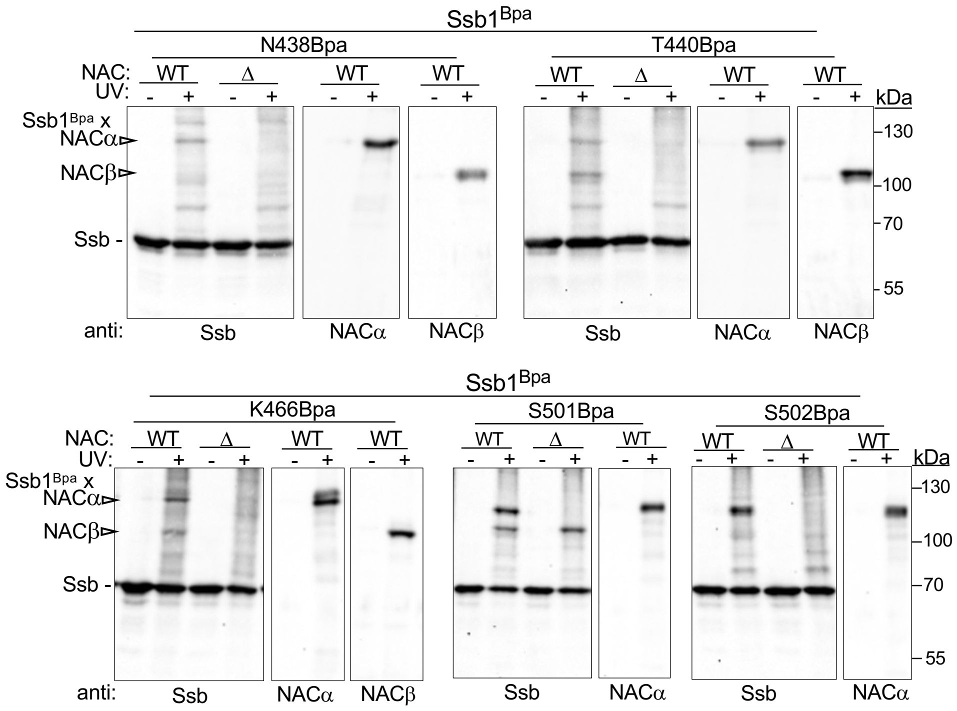


**Figure S5**. Crosslinking of Ssb1$^{Bpa}$ to NAC.

Cells expressing Ssb1 variants with Bpa incorporated at indicated positions were exposed to UV light (+) or left unexposed (-). Crosslinking was analyzed by immunoblotting after SDS-PAGE using antibodies specific for (anti) Ssb, NACα or NACβ, as indicated. Ssb1$^{Bpa}$-NACα/β crosslink products are indicated with arrowheads; noncrosslinked proteins and migration of molecular weight markers (kDa) by dashes. Δ, deletion of *EGD1* gene encoding NACβ.

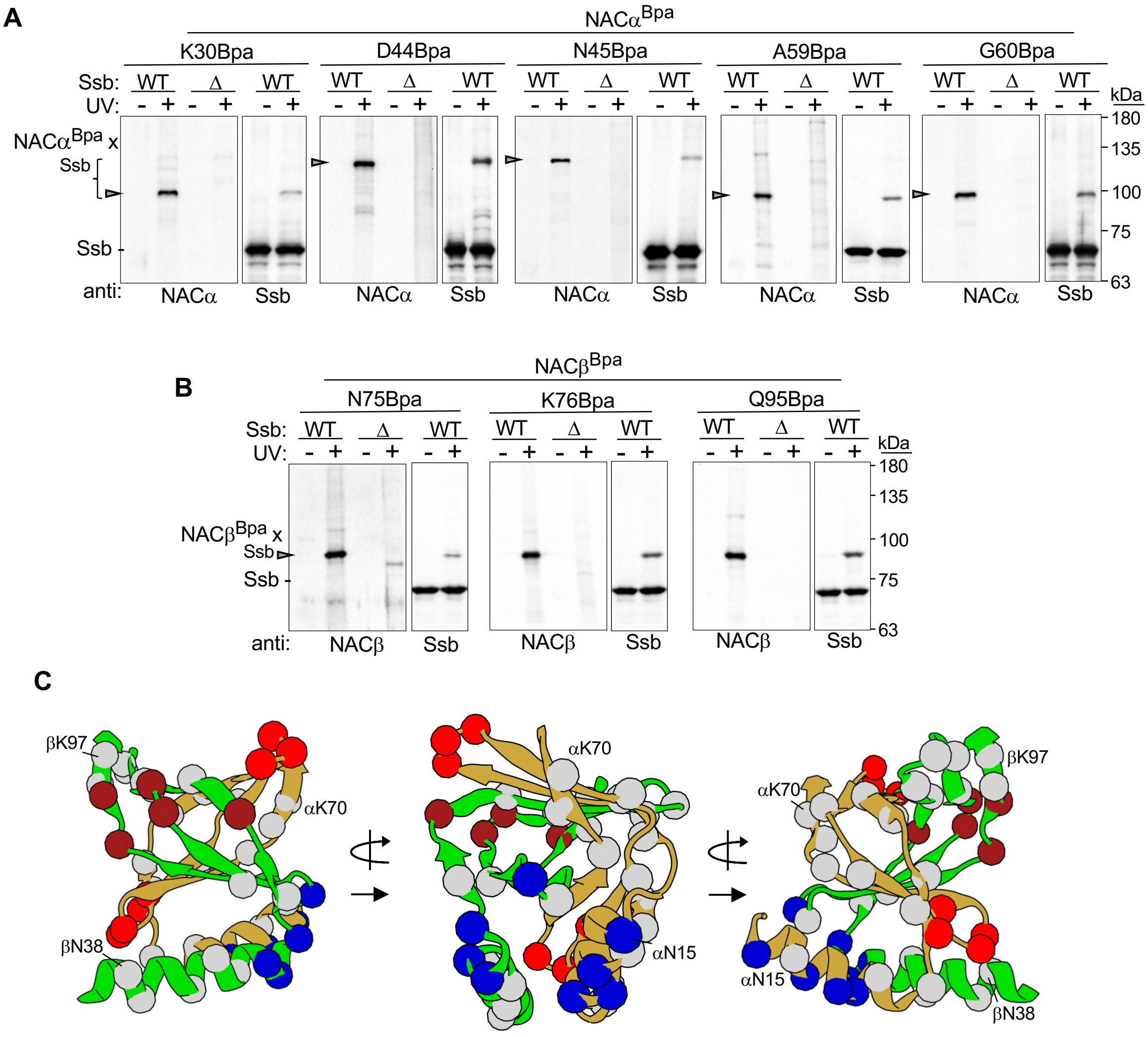


**Figure S6**. Crosslinking of NACα$^{Bpa}$ and NACβ$^{Bpa}$ to Ssb.

(A,B) Cells expressing NACα (A) or NACβ (B) variants with Bpa incorporated at indicated positions were exposed to UV light (+) or left unexposed (-). Crosslinking was analyzed by immunoblotting after SDS-PAGE using antibodies specific for (anti) NACα, NACβ or Ssb as indicated. NAC$^{Bpa}$-Ssb crosslink products are indicated with arrowheads; noncrosslinked protein and migration of molecular weight markers (kDa) by dashes. Δ, deletion of genes encoding Ssb1 and Ssb2. (C) Model of NAC globular domain (10.5452/ma-bak-cepc-0495); α subunit, brown; β subunit, green. Positions Bpa was incorporated and tested for crosslinking to Ssb and Zuo1 are shown as spheres: Ssb positives, red (as shown in Figure 5); Zuo1 positives, blue (as shown in Figure 3); negatives for both Zuo1 and Ssb crosslinking, light gray. NACα$^{Bpa}$ negatives and positives for Zuo1 as in Figure 3 (bold, blue) and positives for Ssb (bold: NACβ, dark red; NACα, bright red ) shown are **K15**, K18, **K19**, **E22**, **L23**, G25, G28, **K30**, Q31, I32, I35, R37, **K43**, **D44**, **N45**, E51, E54, **R57**, **A59**, **G60**, G67, A69, K70. NACβ$^{Bpa}$ negatives and positives as in Figure 3 (bold, blue) and positives for Ssb (bold) shown are N38, K39, K43, Q45, Q47, **A49**, **K50**, **H52**, V54, **N58**, E61, K66, D68, K70, H73, **N75**, **K76**, Q80, **Q84**, H85, **L93**, **Q95**, E96, K97.
